# Host PI3K inhibition via anti-cancer drug alpelisib influences Influenza A non-infectious particles and deletion-containing viral genomes

**DOI:** 10.1186/s12964-025-02598-x

**Published:** 2025-12-19

**Authors:** Ilechukwu Agu, Ivy José, Abhineet Ram, Daniel P. Oberbauer, John G. Albeck, Samuel L. Díaz-Muñoz

**Affiliations:** 1 Department of Microbiology and Molecular Genetics, University of California Davis, One Shields Ave, Davis, CA 95616 USA; 2 Department of Molecular and Cellular Biology, University of California, Davis One Shields Ave, Davis, CA 95616 USA; 3 Genome Center, University of California, Davis One Shields Ave, Davis, CA 95616 USA

## Abstract

RNA viruses can generate “defective” viral genomes during replication, which can interact with standard viral genomes affecting the course of infections. These non-standard viral genomes are related to milder clinical outcomes and are currently being tested as antivirals. Decades of research in influenza have focused on viral mechanisms affecting the production of deletion-containing viral genomes (DelVGs). Based on adaptations of influenza NS1 protein to manipulate host cell metabolism, we hypothesized host metabolic state could also alter the quantity and pattern of deletion-containing viral genomes and the particles that house them. To test this hypothesis, we manipulated host cell anabolic signaling activity and monitored the production of DelVGs and non-infectious particles by two influenza strains, using single-cell immunofluorescence and third-generation sequencing. We show that: 1) influenza infection activates PI3K signaling, with the A/H1N1 strain having roughly double the pAKT levels in single cells as the A/H3N2; 2) alpelisib, a PI3K receptor inhibitor, subverted the ability of both influenza strains to activate PI3K in a dose dependent manner; 3) DelVGs were increased roughly tenfold in polymerase complex segments and ~ 60% in the hemagglutinin segment of A/H1N1 at 20uM of alpelisib; and 4) the A/H3N2 strain did not show changes in DelVG production, but had a modest, statistically significant maximum increase of 11% in non-infectious particles. We find that host cell metabolism can increase the production of non-infectious particles and DelVGs during single rounds of infection, shifting potential interactions among virions. The differential results according to strain and alpelisib concentration suggest future directions examining strain differences in the NS1::p85β virus-host interaction and the specific metabolic state of the cell. Our study presents a new line of investigation into metabolic states associated with less severe flu infection and opens the possibility for potential induction of these states with metabolic drugs.

**Supplementary Information:**

The online version contains supplementary material available at 10.1186/s12964-025-02598-x.

## Introduction

RNA virus infections frequently produce nonstandard viral genomes during replication, which can influence the course of infection through interactions such as complementation and interference [1]. Nonstandard viral genomes have become a recent focus of intense pre-clinical [2–10] and clinical research [11–13], with their antiviral potential holding substantial implications for clinical applications and pandemic preparedness.

Influenza A virus infections are primarily composed of virions that cannot mount a complete infectious cycle, hereafter non-infectious particles. Only 1–30% of virions can propagate fully from cell to cell [14–16] and there are multiple reasons why virions are not fully infectious, such as single nucleotide polymorphisms (SNPs) or faulty protein expression. Virions harboring genome segments with internal sequence deletions [17–19] have been termed defective interfering particles (DIP) when their accumulation diminishes the productivity of Influenza A infections and leads to mild disease outcomes; a phenomenon termed *defective interference* [1, 20–22]. These defective interfering particles do not produce the proteins necessary for a single DIP to complete an infection and relies on coinfection to disseminate [9]. However, upon complementation, the viral genomes with internal deletions can actively interfere with the production of full-length viruses. Recently it has been shown that these “defective” viral segments can actually encode proteins [23] that can also interfere with full length viral replication by directly inhibiting polymerase complex function. Thus, we will refer to these segments containing internal deletions as Deletion-containing **V**iral **G**enomes (hereafter DelVGs, after [24]). This rising relative abundance of DelVGs at the expense of full-length viral genomes [1, 23] has been associated with mild disease outcomes [13]. The flood of complementation-dependent virions sets the stage for interactions that can alter the course of Influenza A pathogenesis, inspiring research into the factors that influence the generation of DelVGs.

Considerable effort has gone into investigating the viral factors that influence DelVG production, such as: high multiplicity of infection, MOI [25],specific mutations of the viral polymerase [13, 26] or matrix genes [27]; and, at a molecular level, polymerase pauses that lead to an internal deletion [18, 28, 29]. Despite much research into the viral factors affecting DelVG production and the effects of DelVGs on the host [30], an almost completely unexplored potential modulator of DelVG production is the host cell. Host cell metabolism and metabolic signaling, can affect progeny virus yield and the severity of infection. For instance, caloric restriction and diet-induced obesity in mice eroded immune response and survivability [31] and in humans, non-obese patient groups exhibit lower viral loads [32, 33]. At a cellular level, reprogramming host tricarboxylic acid cycle with excess malonate diminishes total viral progeny yield in a dose-dependent manner [34], similar to a recent study that increased the TCA cycle in MHV, mouse hepatitis virus [35]. A more recent study in influenza highlights how the TCA cycle is affected in influenza infections [36]. Furthermore, Influenza A has adaptations to steer host metabolism to facilitate productive infection, through specific mutations that affect phosphoinositide-3-kinase (PI3K), a crucial upstream gatekeeper of pro-growth signal transduction networks [37, 38]. A highly-conserved Y89 residue on the influenza NS1 effector protein has a selective and inhibitory interaction with the SH2 domain of host p85β—the regulatory subunit of Class 1a PI3K—which unleashes the catalytic p110α subunit to aberrantly activate PI3K signaling in the absence of a bona fide signaling growth factor like insulin [39, 40]. Thus, the action of NS1 contorts host metabolism into a state characterized by increased pools of the very precursor metabolites [41–43] necessary for uninterrupted biosynthesis of virion components, facilitating pathogenesis [39, 40]. Inhibiting NS1::p85β with ΔNS1(Y89F) expectedly diminishes viral-induced PI3K activation and progeny yield [39]. Because diminished viral yield can result from DelVG accumulation and the inducers of DelVG production remain unknown, it is a reasonable secondary hypothesis that NS1::p85β inhibition—or other form of PI3K inactivation—diminishes viral yield wholly or in part via the induction of DelVG production. However, to our knowledge, the impact of host metabolic signaling on the production of Influenza A non-infectious particles and DelVGs has not been studied.

Given the well-established crosstalk between host metabolism and Influenza A pathogenesis, we hypothesized that disrupting flu-mediated activation of PI3K signaling with alpelisib, —a highly selective small molecule inhibitor of PI3K [44–46]— would increase DelVG and defective virion production. We found that alpelisib treatment overrides virus-induced PI3K network signaling upregulation during infections with both circulating human influenza strains. Alpelisib increased DelVG production in polymerase complex segments at the 20 µM dose in the A/H1N1 strain. Moreover, there was evidence for defective interference in A/H1N1 infections as DelVGs increased while total viral genomes detected decreased. At the virion level, the proportion of non-infectious particles produced by the A/H3N2 strain infections was significantly increased. Collectively, these results suggest that host Class 1a PI3K metabolic signaling receptor inactivation affects the outcome of Influenza A virus infections, steering the population towards more non-infectious particles and increased DelVG production in a strain dependent manner.

## Methods

### Cells and viruses

We obtained MDCK-London cells from the Unites States Centers for Disease Control and Prevention (CDC) Influenza Reagent Resource (IRR). We maintained cells in minimum essential media (MEM) plus 5% fetal bovine serum (FBS). Egg-passaged wildtype A/California/07/2009(H1N1) and A/Texas/50/2012(H3N2) influenza strains were a gift from the lab of Dr. Ted Ross. These initial stocks were double plaque purified in MDCK cells (ATCC/BEI) and propagated thereafter at low multiplicity of infection (MOI = 0.001) in MDCK cells (ATCC/BEI).

### Alpelisib dosing assay

To confirm that alpelisib inhibits PI3K network signaling in MDCK-London cells, we seeded MDCK-London cells overnight at low density in MEM plus 5% FBS media for 24 h into collagen-treated, glass-bottom 96-well tissue culture plates. We then reduced serum in the partially confluent monolayers for 24 h: MEM plus 5% bovine serum albumin (BSA). Three hours prior to conclusion of serum reduction, we spiked a 10 µL pre-treatment of DMSO vehicle control or 21X alpelisib directly into 200 µL of supernatant to reach a 1X concentration. We then mock-infected monolayers with MEM plus 2% BSA and 1% Anti-Anti (Virus Infection Media; VIM) or virus-infected at a multiplicity (MOI) of 1 in VIM; no trypsin was used to limit multiple infection cycles. As part of the inoculation regimen, we spiked 1.9 µL of DMSO or 21X alpelisib into 40 µL of the inoculum supernatant for a 1X concentration, in order to sustain drug effects throughout the virus-monolayer adsorption period. After the 1 h adsorption incubation, we aspirated inocula, washed monolayers and topped with VIM, and we spiked 10 µL of DMSO or 21X alpelisib directly into 200 µL of VIM supernatant for a 1X concentration. After 17 h.p.i., we harvested and titrated supernatants to determine fully infectious (i.e., propagation-capable) and defective (i.e. propagation-incapable) progeny virus yield via our cluster-forming assay (see Methods and Supplementary Materials). We fixed monolayers, conducted immunofluorescence (IF) staining, and imaged to derive cellular-level phospho-AKT signal intensity (see Methods) as a readout for PI3K network signaling activity. We ran three biological replicates of the experiment on different days.

### Single-cell dose response pAKT immunofluorescence quantification

To determine inhibition of PI3K, we sought to measure the downstream effector of PI3K, pAKT (phosphorylated-Serine473-AKT; AKT is another name for Protein Kinase B). At the end of the alpelisib-treated flu infections, we fixed monolayers with 4% paraformaldehyde (PFA). Primary staining was carried out with rabbit monoclonal antibodies targeting pAKT(S473) (Cell Signaling mAb#4060) and mouse monoclonal antibodies targeting Influenza A nucleoprotein (Millipore Sigma MAB8257). Secondary staining was respectively carried out with fluorophore-conjugated goat-anti-rabbit (Thermo Fisher Scientific A-21245) and goat-anti-mouse (Jackson ImmunoResearch 115–645-062) antibodies. We imaged IF-stained monolayers on a Andor Zyla 5.5 scMOS camera and a 20x/0.75 NA objective microscope. Images were then processed to derive cellular-level pAKT, and nucleoprotein signal intensities. The image data were stored as.nd2 files and retrieved using the Bio-Formats toolbox for MATLAB, which can be obtained from www.openmicroscopy.org/bio-formats. Subsequently, a specialized MATLAB cell segmentation pipeline [47] was employed to process the images. Briefly, this pipeline removed background intensity from each image, and then utilized Hoechst 33342 as the basis for image segmentation to mark the nuclei of individual cells. After image segmentation, boundaries (masks) were computationally traced around the nucleus and cytoplasm of each cell (Figure S3.8A), and the average pAKT signal from both regions was summed to derive a value for each cell. The MATLAB pipeline output florescence for each cells in AU (arbitrary units, a measure of signal intensity). To control for any biases in image selection, we randomly subsampled a third of the data set prior to analyses reported; results were comparable with the full data set (see code for details https://github.com/pomoxis/Alpelisib-SIP).

### Cluster-forming assay: titration of fully infectious and defective virions

To determine the titer of fully infectious (i.e., propagation-capable) and defective (i.e. propagation-incapable) virions simultaneously, we developed the cluster-forming assay. This assay combines aspects of the conventional plaque assay with the immunocytochemical staining and microscopy, as done by Brooke et al. [14]. Specifically, the cluster-forming assay employs a low-viscosity overlay medium that remains in a semi-solid state, restricting diffusion of progeny virus to directly adjacent cells, much like a plaque assay. This low viscosity overlay is removable, so that monolayers can be fixed, stained with IF antibodies, and imaged like an immunofocus assay [48]. The basic principle is that virions that were fully infectious would spread from cell to cell, forming clusters of florescence, while virions that were unable to spread would appear as individual foci [14]. We build on previous similar assays [14] by automating the image processing and analysis to identify productive and abortive infections.

We briefly describe this assay below and provide detailed methods in Supplement: We seeded MDCK-London cells overnight (24 h) at high density in MEM plus 5% FBS media into collagen-treated, glass-bottom 96-well tissue culture plates. We inoculated the confluent monolayers with serial dilutions of virus-borne supernatant and incubated for 1 h to facilitate virus-monolayer adsorption, after which we aspirated the inoculum, washed monolayers with VIM, and overlaid monolayers with medium-viscosity culture medium (VIM plus 4% carboxymethyl cellulose and 1 ug/mL TPCK-Trypsin). At 11 h.p.i. we aspirated overlay medium and fixed monolayers with 4% PFA. We stained fixed monolayers with fluorophore-conjugated ICC/IF antibodies targeting Influenza A nucleoprotein (NP) and counter-stained with Hoechst. We imaged IF-stained monolayers on a fluorescence microscope to reveal the number of fully infectious (i.e. infections that spread from cell-to cell) and incompletely infectious (i.e., infections that crossed a productivity threshold within cells, but did not spread from cell–cell) events, respectively depicted by a cluster of infected cells (productive clustering unit/PCU), or solitary infected cells (non-clustering unit/NCU) (Fig. 4, Figure S3.1–3.5). PCU’s are defined as > 1 infected cell, including 2-cell clusters because the key factor we aimed to distinguish was “spread” past the original infected cell within the assay window (12 h). Our guiding design principle was to cordon—or mask—nucleoprotein fluorescence signals (GFP) in the IF image as independent infection events, then overlay said mask with the host nuclei segmentation Hoechst signal to reveal the number of cells each infection event had spread to. We began stepwise assembly of masks around the GFP signals (Fig. 4B-C) by binarizing IF images with the *imbinarize* function to make object detection possible, followed by the removal of small, noisy pixels with *bwareaopen*. Masks were sequentially dilated then filled with *imdilate* and *imfill* functions respectively to smoothen them out and ensure they did not contain holes. To finish the mask assembly, masks were eroded with *imerode* to undo the signal expansion done in the dilation step (Fig. 4C). Undesired masks were filtered out by thresholding the min/max mask area and removing masks that did not contain any nuclei, leaving bona fide infection events—or clusters—that are counted and assigned a unique identity number, a *clusterID* (Fig. 4D). To quantify PCUs and NCUs, we utilized MATLAB's image processing toolbox. Immunofluorescence images were processed to create object masks for each unit, and nuclear segmentation was performed via the Hoechst signal (Figure S3.8). Masks were refined and filtered, and the number of cells within each unit was determined (Figure S3.8). The R Programming Language was employed to classify clusters as PCUs or NCUs based on size, followed by calculation of NCU titer and proportion.

### Viral genome sequencing by nanopore long-read sequencing

To derive the genomic sequences of progeny Influenza A virus from our treatments, we began by isolating viral genomic RNA from 100 µL of treatment group supernatants (Zymo Research, Quick-DNA/RNA Viral MagBead kit R2140). Next, we used a 2-cycle RT-PCR reaction (Invitrogen SuperScript™ III One-Step RT-PCR System with Platinum™ Taq DNA Polymerase kit 12574026) to reverse transcribe viral genomic RNA into the first cDNA strand (1st PCR cycle), and then synthesize the second cDNA strand (2nd PCR cycle). The RT reaction to produce the first cDNA strand was primed with a 45 bp forward primer (Integrated DNA Technologies) that included a complementary sequence to the uni12 region shared by all flu genomic segments (12 bp), flanked with a unique molecular identifier (UMI) sequence (12 bp) and a landing pad sequence for downstream barcoding primers (21 bp): fwd 5’-TTTCTGTTGGTGCTGATATTGNNNNNNNNNNNNAGCRAAAGCAGG-3'. The PCR reaction to generate the second cDNA strand was primed with a 47 bp reverse primer that included a complementary sequence to the uni13 region shared by all flu genomic segments (13 bp), flanked by a UMI sequence (12 bp) and the barcoding primer landing pad sequence (22 bp): rev 5'-ACTTGCCTGTCGCTCTATCTTCNNNNNNNNNNNNAGTAGAAACAAGG-3'. We used AMPure XP beads (Beckman Coulter, AMPure XP A63881) with manufacturer's instructions to remove excess primers, followed by a 17-cycle amplification PCR (Invitrogen, Platinum SuperFi Master Mix 12358–050) of the umi-tagged reads with barcoding primers (Oxford Nanopore Technologies, PCR Barcoding Expansion 1–96 kit EXP-PBC096). The low number of cycles was designed to minimize PCR duplicates. We then pooled 60 ng of barcoded amplicons from each sample, cleaned and concentrated this pooled sample (Zymo Research, Select-A-Size DNA Clean & Concentrator D4080), and prepared a sequencing library in accordance with manufacturer instructions (Oxford Nanopore Technologies, Ligation-Sequencing-Kit-V14 SQK-LSK114). We loaded the pooled libraries into an R10 flow cell connected to a MinION MkIB device and ran a 72 h sequencing protocol from the MinKNOW control software. Upon sequencing run termination, we used the Guppy basecaller software to barcode-demultiplex sequenced reads into their respective treatment groups. The advantage of this sequencing approach for identifying DelVGs, is that it allows for the sequencing of intact amplicons, regardless of length. Unlike short-read sequencing platforms, genomes are not fragmented, so DelVGs can be directly captured for entire genome segments in single sequencing reads, unlike previous established approaches which infer DelVGs based on coverage differences.

### Classification and quantification of DelVGs and full-length viral segments

We describe the pipeline in brief below; details are in the GitHub code repository (https://github.com/pomoxis/alpelisib-SIP). Demultiplexed amplicon sequences underwent quality control pre-processing prior to deduplication into representative sequences, after which representative sequences were classified into subgroups for DelVGs and full-length, standard viral genomes (SVG).

#### Quality control

Our sequencing library preparation strategy began with a 1-cycle each RT-PCR then PCR addition of 12 bp-long UMI sequences to the 3’ and 5’ termini of viral genomic RNA, followed by PCR addition of sequencing barcodes to both termini:

- 5’-barcode—spacer—landing.pad—UMI—uni12—*locus*—uni13—UMI—landing.pad—spacer—barcode-3’

For quality control, we trimmed off barcode and barcode landing pad regions with C*utadapt*, then used *Cutadapt* once more to filter-in only amplicons with a 12 bp-long UMI region. Finally, we confirmed the presence of well-formed uni primer regions in the filtered amplicons before advancing to UMI deduplication.

#### UMI deduplication

We used *UMI-Tools* to sequentially group PCR duplicates by UMI, and then collapse them into a single representative read. In the final quality control step, we trimmed the uni primer region off representative reads with *Cutadapt*. By integrating UMI-deduplication into our workflow, we've mitigated the impact of PCR amplification bias on sequencing depths. Consequently, our delVG and SVG count data represent a quantitative measurement of the abundance of RNA molecules (genome segments) from which the amplicons were derived.

#### DelVG characterization

UMI-deduplicated fastq files containing read sequences were processed with the Virus Recombination Mapping (*ViReMa*) software to identify recombination events per genomic read using the following parameters [49, 50, 51]:

*--*Seed 25 --MicroInDel_Length 20 --Aligner bwa --ErrorDensity 1,25

Additionally, the *-ReadNamesEntry* switch was included in a separate ViReMa run of the same dataset in order to assign read name information to each recombination, which allowed us to collapse deletion events with the same read name into a single delVG observation with in-house Bash and AWK scripts:

*--*Seed 25 --MicroInDel_Length 20 --Aligner bwa --ErrorDensity 1,25-ReadNamesEntry

#### Full-length viral genome characterization

To characterize SVGs, we began by using the *bwa* alignment tool to determine the properties of reads and their alignment to the reference genome; this information is captured in the bitwise FLAG field (column 2) of the output SAM file. Next we used the *AWK* program to select only reads with proper alignment to the forward and reverse strands of reference genome—bitwise FLAGs 0 × 0 (0) and 0 × 10 (16) respectively—and used *AWK* yet again to filter reads that were within ± 100 bp the length of the reference genomic segment.

### Statistics

In general, we relied on linear or linear mixed models to test significance between treatments using base R and the nmle packages, respectively. Owing to the intrinsic heterogeneity of flu infections [52, 53], we included biological replicates as a random factor, unless otherwise indicated. We tested alpelisib inhibition of PI3K network signaling and its dose dependence, using a linear model and Dunnett’s contrasts. We tested influenza activation of PI3K network signaling activity using ANOVA and Tukey’s HSD, to test for differences among strains and the mock infection. We tested whether alpelisib affects the production of defective virions, by testing for differences in the proportion of NCU’s according to each alpelisib dose using a linear mixed model. We similarly tested for differences in the total viral particles detected by the cluster-forming assay. Finally, we tested whether alpelisib increases DelVG production by examining the proportion of DelVGs in each alpelisib concentration using a linear mixed model that controlled for viral genome segment identity, as these have documented differences in the production of DelVGs during infection. Code for statistics and analyses is posted on GitHub (https://github.com/pomoxis/alpelisib-SIP).

## Results

### Influenza A infection activates PI3K network signaling activity with strain-specificity in MDCK cells

**Fig. 1:**
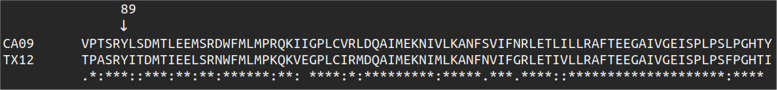
CA09 and TX12 have the highly conserved Y89 residue that is necessary for Class 1a PI3K activation. Multiple sequence alignment of CA09 and TX12 NS protein amino acid sequences. * indicates a fully conserved residue; : denotes residues that are different but share similar chemical properties, a conservative substitution; . denotes a semi-conservative substitution

Class 1a PI3K signaling in host cells can be activated by several Influenza A strains, including A/WSN/1933(H1N1), A/Udorn/72(H3N2), A/Victoria [8, 32,], A/Puerto Rico/8/34(H1N1) [39, 40, 54], and an unspecified 1918 pandemic H1N1 strain [55]. The effector domain of the viral NS1 effector protein is the molecular activator of the PI3K signaling cascade [39, 55, 56], and different influenza strains have been shown to differentially activate PI3K signaling in a tumorigenic cell line [56]. To avoid confounding our results with the aberrant PI3K signaling typical in cancer cells [57–60], and considering the strain-specific differences in the NS1 effector domain (Fig. 1), we needed to ensure our chosen strains could activate PI3K network signaling in a non-tumorigenic MDCK cell line (but see [61]) within the experiment window. Thus, we quantified pAKT activity at the single cell level and found strong evidence that alpelisib inhibited PI3K network signaling with a clear dose dependent decrease in pAKT activity (Adjusted R^2^ = 0.1672, *p* < 0.00001, Figure S1 and Extended Results in Supplementary Materials).

**Fig. 2:**
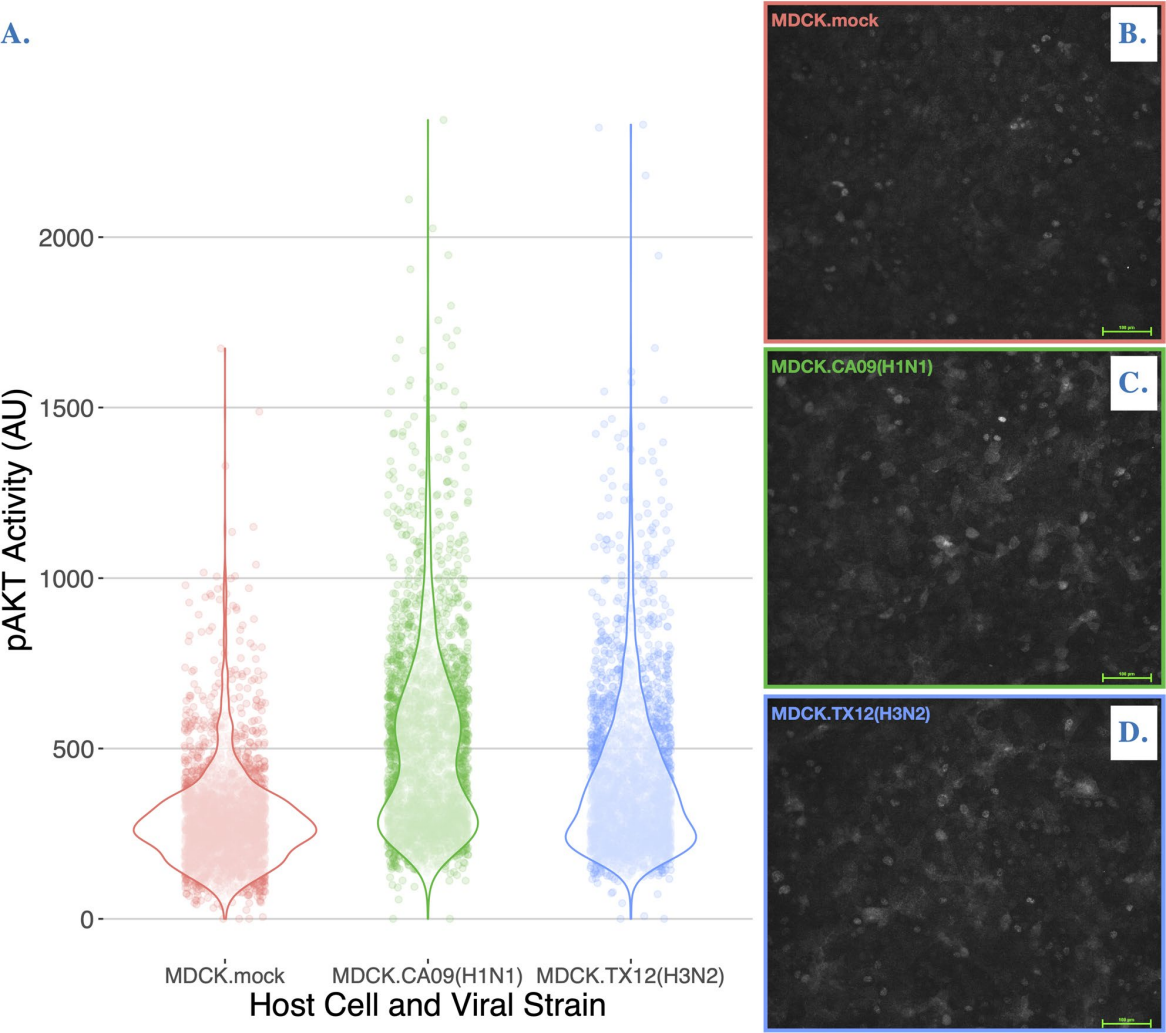
Differential activation of pAKT activity by Influenza A infection. **A** pAKT activity (AU) of influenza-infected MDCK-London cells. Individual points represent individual cells from three biological replicates. Violin plots overlaid depict the frequency of points using density curves. **B-D** Representative images of each of the three corresponding conditions

We measured pAKT in CA09 and TX12 infections compared to a mock infection, all three conducted in three biological replicates. Both viral infections upregulated pAKT compared to mock infection in a statistically significant way (ANOVA Adjusted R^2^ = 0.2428, *p* < 0.00001), with CA09 and TX12 increasing the AU by an average of 205.861 and 106.886 respectively (Tukey Contrasts *p* < 0.00001). TX12 upregulation of pAKT was on average −98.976 AU less than CA09 and this effect was statistically significant (Tukey Contrasts *p* < 0.00001) (Fig. 2). The results were qualitatively and quantitatively very similar if we subsampled a third of the data set – we used the entire data set in the statistics and figure. In addition to confirming PI3K activation by CA09, we discovered this trait in TX12 and demonstrated the differential dysregulation of PI3K signaling by both strains in a non-tumorigenic cell line.

### Alpelisib pre-treatment is sufficient to subvert PI3K network signal restoration by Influenza A

**Fig. 3:**
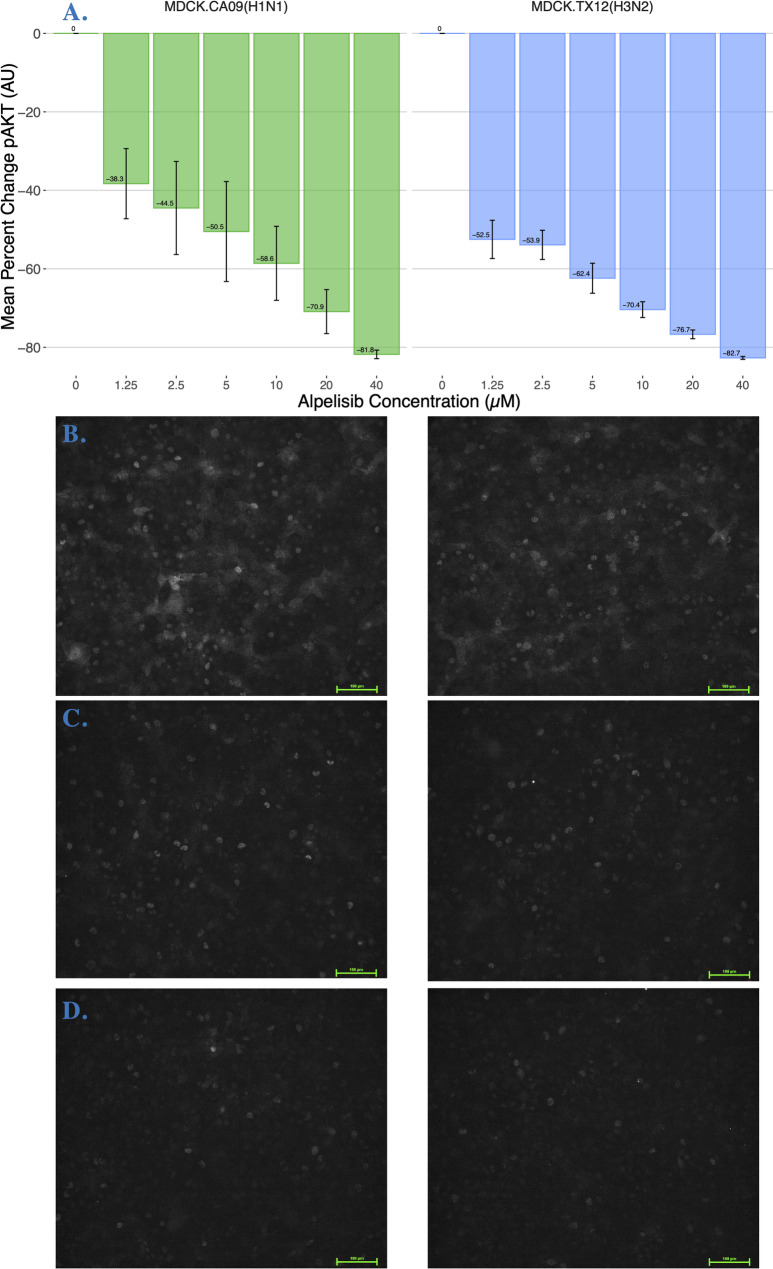
Alpelisib significantly inhibits pAKT activity during Influenza A viral infection. **A** Mean percent change in pAKT activity (AU) of MDCK-London cells pre-treated with increasing concentrations of alpelisib and then infected with either CA09 or TX12; 0 µM alpelisib treatment group received vehicle solvent (DMSO). Bars represent the average of three biological replicates and error bars depict the SEM. **B-D** Representative images of pAKT signal intensity (gray) for select doses of alpelisib, CA09 on the left, TX12 on the right under their respective plots: **B** 0 µM alpelisib, vehicle-treated; **C** 10 µM alpelisib; and (**D**) 40 µM alpelisib

To determine PI3K-AKT signaling outcomes under the competing influences of influenza and alpelisib, we measured pAKT activity in cells pre-treated with increasing concentrations of alpelisib and then infected with either CA09 or TX12. We found that alpelisib completely subverted the observed viral PI3K upregulation in a dose-dependent fashion (Fig. 3, Supplementary Figure S2); in both CA09 (Adjusted R^2^ = 0.3313, *p* < 0.00001) and TX12 (Adjusted R^2^ = 0.2725, *p* < 0.00001) strains at alpelisib concentrations from 1.25–40 uM. Each 1 µM increase in alpelisib led to a decrease of 6.4280 and 5.9940 AU in CA09 and TX12 infections respectively. We note that the qualitative pattern and magnitude of the decrease in AU per µM of alpelisib for cells infected with either strain was strikingly similar in uninfected cells (−4.5342 AU). We confirmed that alpelisib inhibits PI3K network signaling in the MDCK-London cell line we used (Supplementary Figure S1).

### The cluster-forming assay can titrate non-infectious influenza a particles

**Fig. 4:**
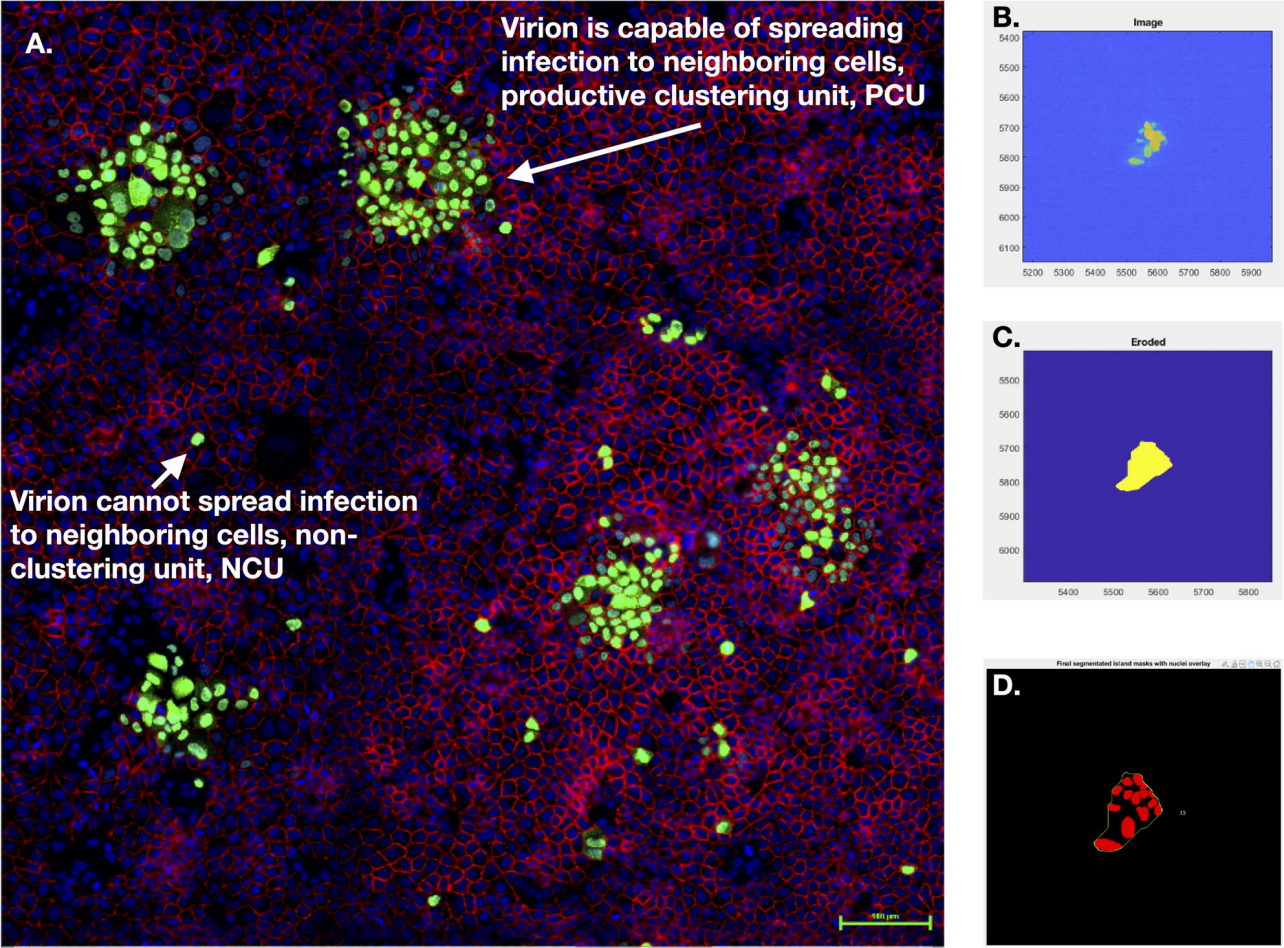
The cluster-forming assay can simultaneously titrate virions that mount productive infections and propagation-incapable virions that do not complete the infectious cycle. Influenza A Virus infection of MDCK-London cells showing productive and abortive infections. Green/GFP – **A**/California/07/2009 nucleoprotein; Blue/Hoechst – MDCK-London nucleus; Red/Cy5 – MDCK-London E-cadherin. **B-C** Stepwise assembly of a mask around the nucleoprotein GFP signal in a productive clustering unit (PCU); starting from the initial cluster-forming assay IF image (**B**) down to final erosion (**C**). **D** segmentation-mask overlay to size (i.e. number of cells) a PCU

We developed the cluster-forming assay to simultaneously titrate fully infectious and propagation-incapable particles by combining elements of the conventional plaque assay [62] and immunofocus assay [48]. While the plaque and immunofocus assays respectively use solid or liquid overlay media to sustain inoculated monolayers for the duration of the assay, the cluster-forming assay employs a medium-viscosity overlay that remains semi-solid [63]. This medium restricts viral diffusion to neighboring cells much like a plaque assay, but can be removed for fixation, staining, and imaging of monolayers akin to the immunofocus assay (see Methods). The cluster-forming assay yields immunofluorescence (IF) images where each infection event appears as either a cluster of infected cells (productive clustering unit, PCU) or solitary infected cell foci (non-clustering units, NCU) (Fig. 4, Supplementary Figures S3-36). PCUs represent a productive infection mounted by a single fully infectious virus particle (analogous to plaque forming units, PFU), whereas NCUs represent self-limiting infections mounted by propagation-incapable viral particles.

**Fig. 5:**
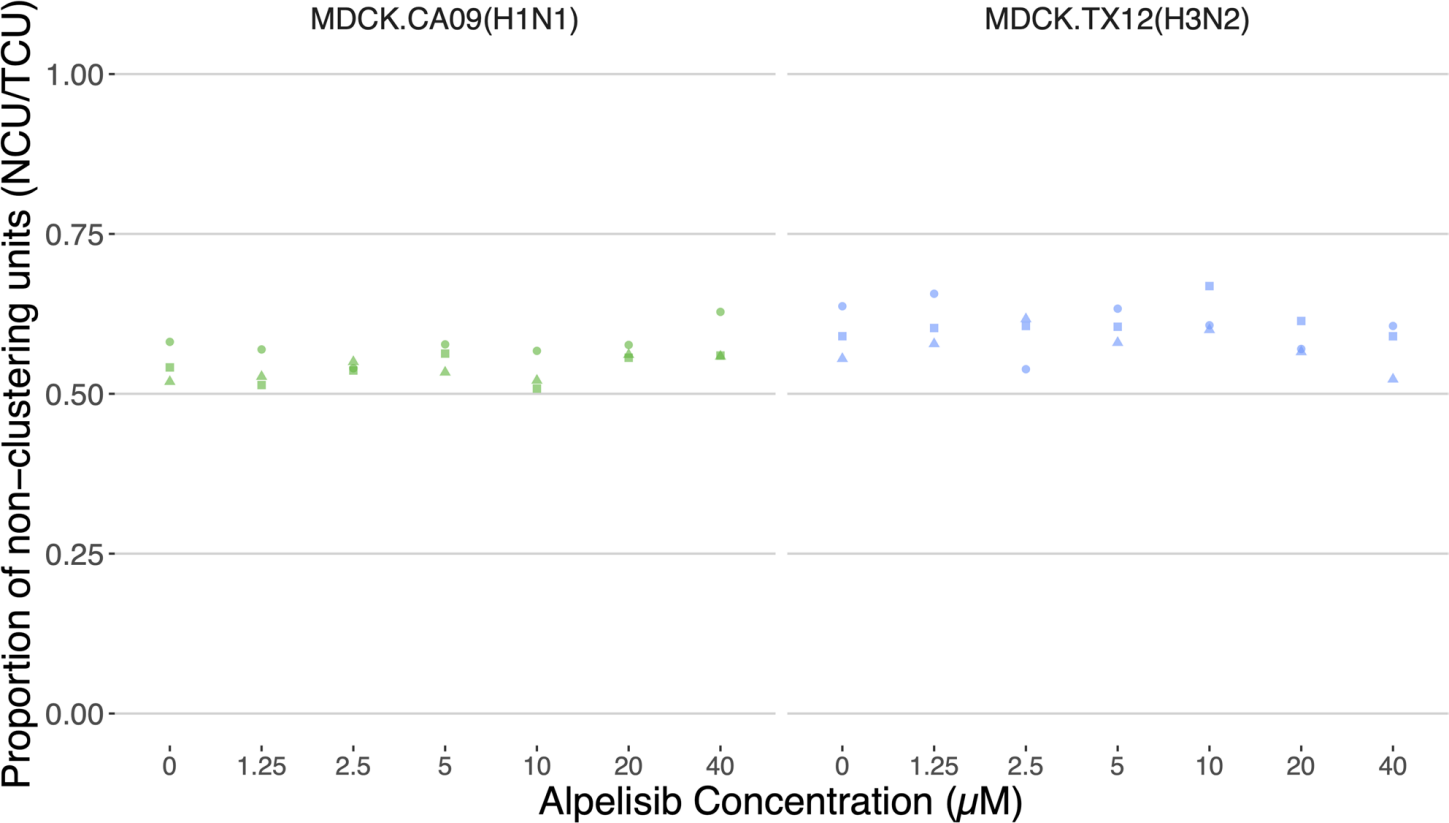
The cluster forming assay is highly reproducible. The proportion of non-clustering units (over total clustering units, TCU, the total infection events including clusters and single positive cells) titrated from supernatants of 18 h CA09 and TX12 infections of cells pre-treated with different concentrations of alpelisib. Each point is a technical replicate; i.e. a titration of the same infection supernatant. The 0 µM alpelisib treatment group received vehicle solvent (DMSO)

To count PCUs and NCUs, cluster-forming assay IF images (Fig. 4A) were put through an automated image analysis pipeline we developed using MATLAB's image processing toolbox (see Methods). The cluster-forming assay is a highly reproducible (Fig. 5) improvement of the conventional immunofocus and plaque assays that provides increased resolution of viral infectivity. In addition to quantifying fully infectious particles—as was possible with a conventional plaque assay—it is now possible to simultaneously quantify particles that are propagation incompetent in the same sample with high-throughput. This is significant because access to two sub-populations of infectious viral particles makes it possible to determine total infectious particles, and thus relative abundances as well as; both of which are indispensable metrics for the quantitation of viral interference within the host.

### Alpelisib affects production of non-infectious particles early in Influenza A infection

**Fig. 6:**
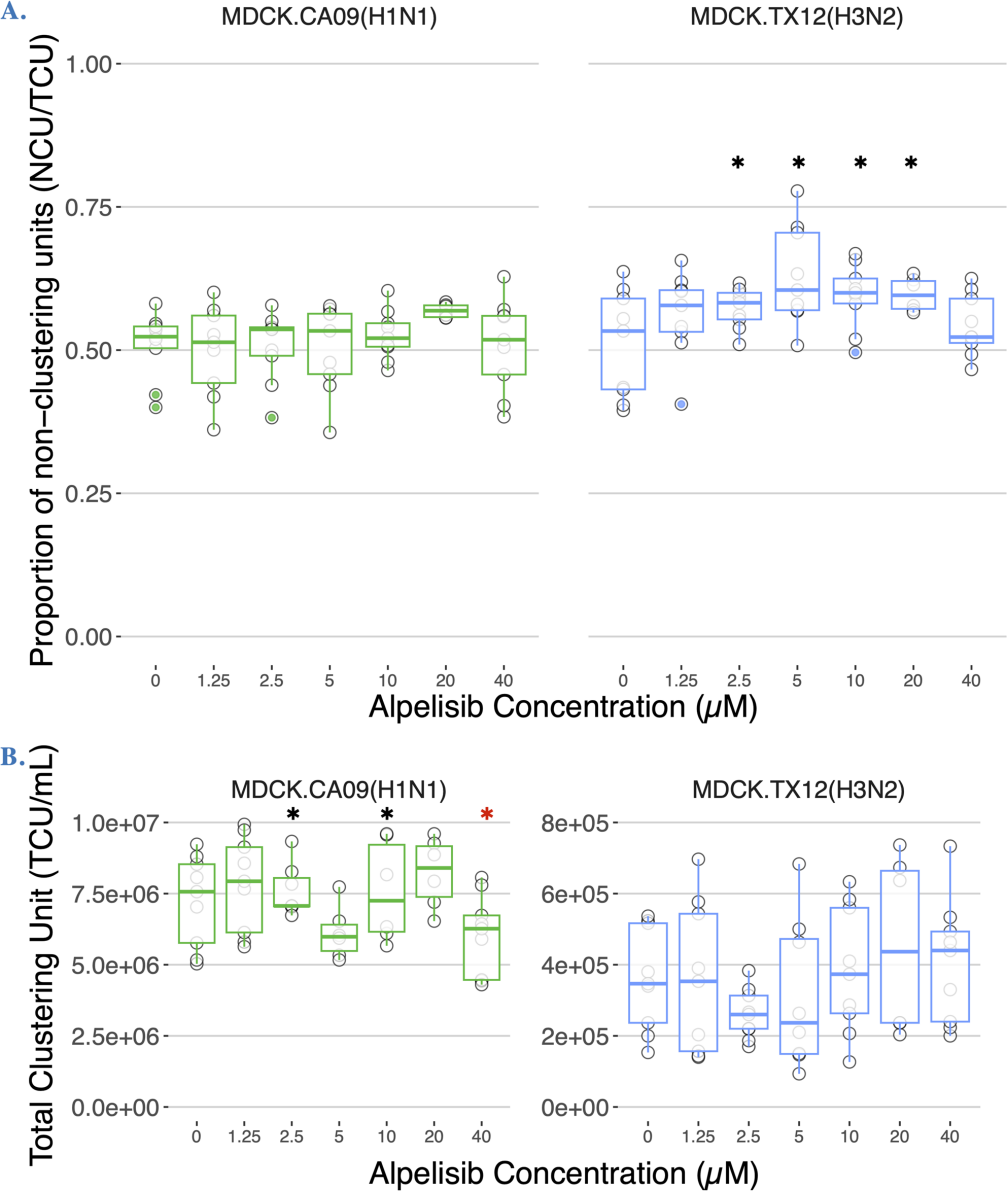
Alpelisib can affect the proportion of non-infectious particles, as well as the total particle yield in a strain dependent manner. Proportion of (**A**) non-clustering units (NCU) and (**B**) concentration of total clustering units (TCU/mL) in CA09 and TX12 at 18 h.p.i. under different concentrations of alpelisib; no trypsin. 0 µM alpelisib treatment group received vehicle solvent (DMSO). Boxplots summarize data from three biological replicates. Histogram colors denote the strain. Black asterisks denote statistically significant increases, red asterisks denote statistically significant decreases. Alpelisib had the most marked effects on the proportion of non-infectious particles in TX12 (H3N2) (**A**, Right panel) and on total particles in CA09 (H1N1) (**B**, Left panel)

To determine whether alpelisib pre-exposure of cells affected CA09 or TX12 infection, we used the cluster-forming assay to screen a broad dosage range of alpelisib pre-treatment concentrations (Fig. 6). Specifically, we sought to determine if alpelisib pre-treatment (0, 1.25, 2.5, 5, 10, 20, and 40 µM) affected the production of defective/non-infectious particles, reported here as the relative abundance or proportion of NCUs. We conducted Linear mixed-effects modeling (nmle) to analyze each alpelisib treatment as a factor because a dose-dependent response was not found for either infection. Alpelisib pre-treatment significantly altered the proportion of NCUs in TX12 (*p* < 0.0001), but in CA09, no treatment was significantly different against the control. In TX12, concentrations of 2.5, 5, 10, and 20 µM alpelisib increased the percentage of NCUs by 6.37%, 11.946%, 8.511% and 6.70% respectively, and these effects were statistically significant (all *p* < 0.0388).

We also examined whether alpelisib pre-treatment affected the total viral yield, here measured by total clustering units (TCU). In CA09 infections, alpelisib was a statistically significant factor affecting TCUs (Adjusted R^2^ = 0.8247, *p* < 0.00001); alpelisib concentrations of 2.5, 10, and 40 µM were significantly different from control, changing TCUs by an average of 1.21 × 10^6^, 1.35 × 10^6^, and −1.20 × 10^6^ TCU/mL, respectively. These values represent *increases* of 16.67% (2.5 µM) and 18.66% (10 µM), as well as a *decrease* of 16.56% in the 40 µM treatment. In TX12 infections, alpelisib was not a statistically significant factor in explaining TCUs (*p* < 0.0632), and there were no significant differences between alpelisib treatments.

### Alpelisib increases deletion-containing viral genome and total viral genome production early in Influenza A infection

We found alpelisib caused changes in infectiousness at the particle level, however the cluster forming assay does not identify the underlying genetic causes of changes in infectiousness. To specifically investigate whether alpelisib caused an increase specifically in DelVGs, we measured DelVGs by sequencing viral supernatants from the same infections that were used in the cluster-forming assay (see Figs. 5 and 6). Two MinION flow cells yielded a total of 27.42 M reads. After quality control for well-formed amplicons (perfect 12nt UMIs, influenza A-specific terminal uni12/13 regions) and de-duplicating unique molecular identifiers (UMI) we obtained 120,652.10 ± 70,902.90 reads per infection (CA09: 148,943 ± 78,828; TX12: 92,362 ± 49,104). We note that these read counts are produced from de-duplicated reads, reflecting an estimate of the RNA genome content whether deletion-containing or not (total viral genomes, TVG) in the supernatants. The deletions we detected were comparable to published studies (example in Figure S5) and in brief, resulted in very large deletions in the polymerase segments, as much as ~ 93% of the genome segment and, on average, roughly 46% across all unique deletions (PB2 = 1077 ± 571, *n* = 131066; PB1 = 1229 ± 675, *n* = 36039; PA = 1155 ± 575, *n* = 232191). This distribution resulted in roughly in an average of 609 bp being retained from the 5’ end and 532 bp being retained in the 3’ end. The average deletion size was somewhat smaller in the antigenic segments (HA ~ 29%; NA ~ 43%) and smaller still in the remaining segments (NP ~ 17%; NS ~ 14%; M ~ 23%. The distribution of deletion sizes is presented in Figure S4.

**Fig. 7:**
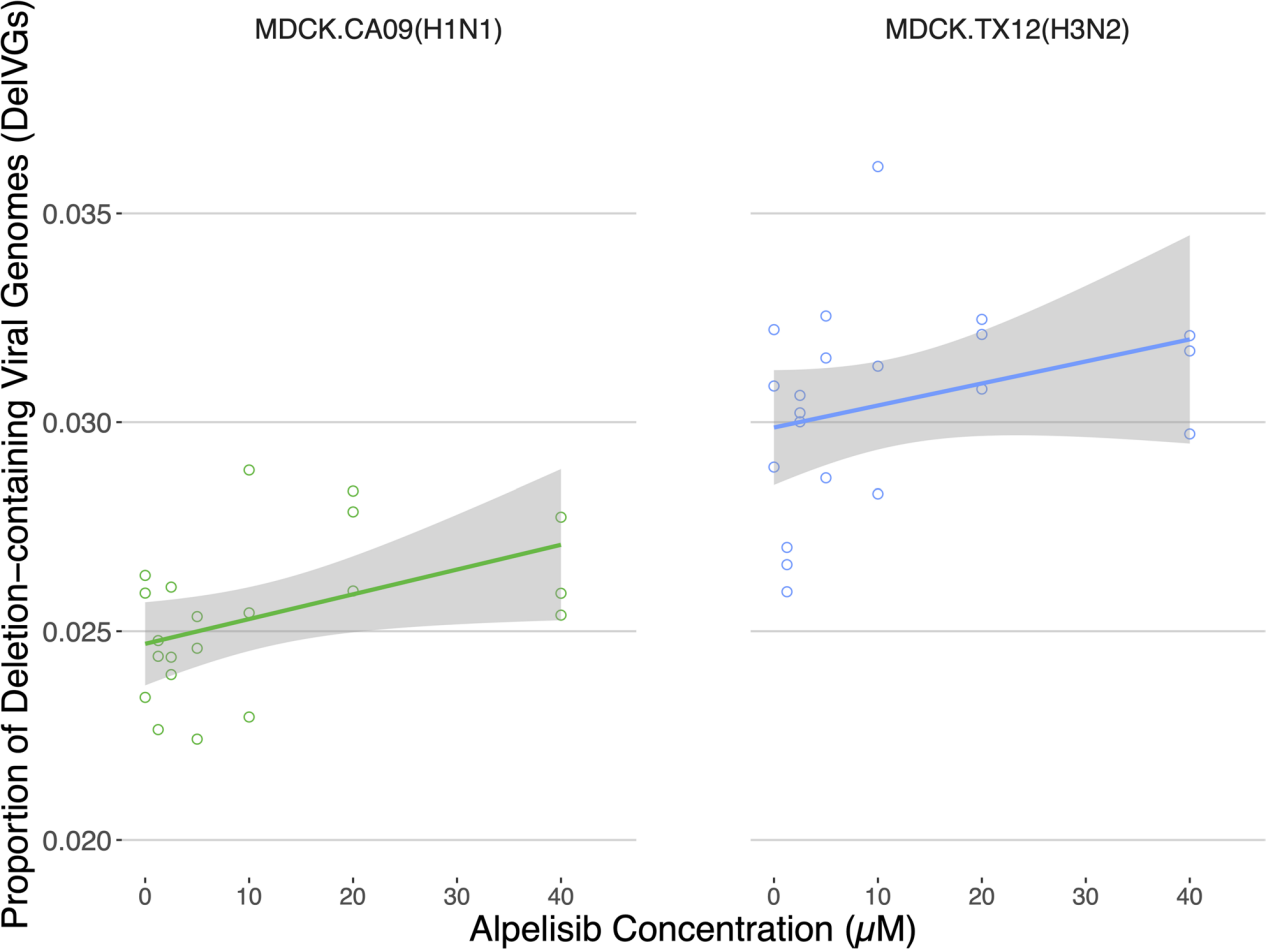
Alpelisib pre-treatment of cells increases the proportion of deletion-containing viral genomes. Overall proportion of DelVGs (i.e. total DelVGs regardless of segment origin) as a function of concentration of alpelisib pre-treatment. The CA09 regression (Left panel) is statistically significant (*p* = 0.008), while TX12’s (Right panel) is not (*p* = 0.1907). Three independent infections with CA09 and TX12 per concentration at 18 h.p.i. under different concentrations of alpelisib pre-treatment; no trypsin. 0 µM alpelisib treatment group received vehicle solvent (DMSO)

To determine if alpelisib pre-treatment of cells increased DelVG production, we tested for differences in the proportion of DelVGs between the mock-infected control and a wide range of alpelisib concentrations. The vehicle-treated control yielded 3903.67 ± 995.55 DelVGs for CA09 and 3601.67 ± 577.42 for TX12 as detected by ViReMa, which respectively represents 2.52% and 3.07% of total viral genomes (154584, 118207). The overall proportion of DelVGs increased as a function of increasing alpelisib concentration in CA09 infections (Adjusted R^2^ = 0.400, *p* = 0.008; Fig. 7, Left). Alpelisib explained 19.96% of the variation in the proportion of DelVGs, with each 1 µM increase of alpelisib increasing the total proportion of DVGs by 0.005929%. Infections with TX12 showed this increasing trend, however the model was not statistically significant (Adjusted R^2^ = 0.1037, *p* = 0.1907, Fig. 7, Right).

Influenza viral genome segments have known variation in their propensity to generate DelVGs. In particular, the polymerase complex genes (PB2, PB1, and PA) are known to generate most of the DelVGs in a given influenza infection [19, 22]. Thus, we made linear mixed models for each segment, separating into strain specific models, if strain was found to be a significant predictor. We examined differences in the proportion of DelVGs treating each concentration as a factor, as there was not a dose-dependent effect per-segment. We report all statistically significant results in Table S1 (blue shading) and the full results are in the code on the GitHub Repository (https://github.com/pomoxis/alpelisib-SIP).

In CA09 infections, all statistically significant increases in DelVG proportion were found at the 20 µM pre-treatment concentration for segments PB1, PA and HA, with respective increases of 0.2473, 0.3737, and 0.0579 proportion units relative to the control mock infections. The CA09 strain has been documented to have higher DelVG production in the HA segment compared to other strains [24]. For context, in vehicle-treated infections polymerase segment DelVGs produced ranged from an average of 3.11%—4.40% of total viral genomes, which increased to 24.67%—40.48% in infections with 20 µM of alpelisib. In TX12 infections, the only statistically significant changes from the control were in segments HA and M, consisting of decreases in DelVG’s of less than 1%. These changes occurred across a broader range of concentrations for the HA segment (1.25, 2.5, 5, 10 µM), than for the M segment (1.25 µM).

**Fig. 8:**
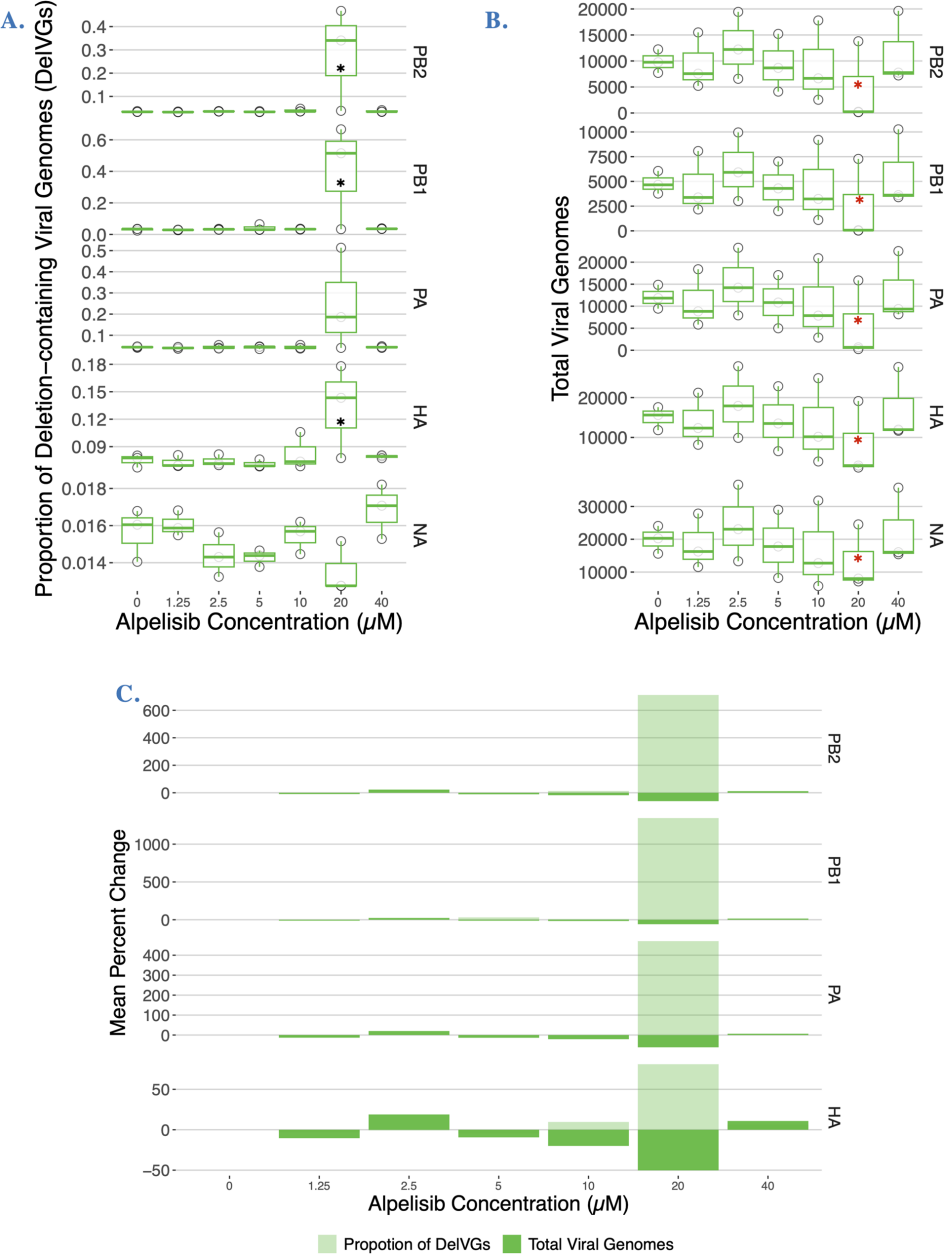
Alpelisib treatment of cells increases the proportion of deletion-containing viral genomes and decreases total viral genomes in CA09(H1N1pdm) infections. **A** Per-segment proportion of Deletion-containing Viral Genomes (DelVGs) at 18 h.p.i. under different concentrations of alpelisib; no trypsin. Black asterisks indicate statistically significant increases. **B** Per-segment Total Viral Genomes (TVG) at 18 h.p.i. under different concentrations of alpelisib; no trypsin. Red asterisks indicate statistically significant decreases. **C** Average percentage change in Deletion-containing Viral Genomes (DelVGs, light green) and Total Viral Genomes (TVG, dark green) under different concentrations of alpelisib. Only segments with statistically significant results are shown. The 0 µM alpelisib treatment group received vehicle solvent (DMSO). Figures depict data from three biological replicates

We also analyzed the number of total viral genomes produced at different alpelisib concentrations using models for each genome segment. We found statistically significant decreases in CA09 infections at the 20 µM concentration (Fig. 8A) in all three polymerase segments and both antigenic segments. Specifically, segments PB2, PB1, and PA had decreases of −5173.3, −2371, and −6456 total viral genomes, respectively, whereas HA and NA showed decreases of −6840 and −6758 total viral genomes, respectively (Fig. 8A; Table S1, gray shading). In TX12 infections, there were no statistically significant changes in TVGs according to alpelisib dose.

Collectively, these results suggest that alpelisib treatment of CA09 infections at 20 µM *increases* DelVG production and *decreases* total viral genomes in two of the polymerase complex segments (PB2, PB1) and the HA segment (Fig. 8C), a signature of defective interference. Statistically significant changes in TX12 DelVGs were of small magnitude and total viral genome production did not show statistically significant differences across alpelisib doses. Furthermore, the overall proportion of DelVGs (regardless of segment) shows a dose-dependent increase as alpelisib concentration increased in CA09 infections (Fig. 7). This same trend was evident in TX12 infections, but the linear regression was not statistically significant.

## Discussion

To investigate the dependence of influenza progeny infectivity on host cell metabolic signaling, we reprogrammed host PI3K network signaling during flu infection with alpelisib, monitored the intervening metabolic state with cellular-level resolution immunofluorescence microscopy, and determined the change in proportion of non-infectious progeny virus with our newly developed cluster-forming assay. The proportion of non-infectious particles in a flu infection is emerging as a crucial determinant of pathogenic outcomes, and non-infectious particles are being directly used as antiviral treatments [2–4, 6–10]. We established that host cell PI3K network signaling activity can influence the proportion of non-infective particles produced by influenza in two strains. First, we found that alpelisib treatment keeps PI3K-AKT signaling pathway activity suppressed during Influenza A infection in MDCK-London cells (Fig. 3), counteracting influenza’s upregulation of PI3K signaling (Fig. 2; [8, 32,]. As predicted by our hypothesis, we saw several effects of alpelisb on DelVGs and non-infectious particle production. Notably, the most striking effects were in the CA09 strain at 20uM alpelisib, where the *abundance* of DelVGs increased roughly tenfold in polymerase complex segments and ~ 60% in the hemagglutinin segment (Fig. 8A) and we also found accompanying effects on the *type* of DelVGs produced (Figure S5, S5). These results were accompanied by a decrease in the total viral genomes quantified (Fig. 8B-C), suggesting these DelVGs were acting as defective interfering genomes. The TX12 strain, which was less capable of activating the PI3K pathway, did not show changes in DelVG production under alpelisib treatment, but had modest increases in non-infectious particles over multiple concentrations (Fig. 6). In sum, we find that it is possible that host cell metabolism can increase the production of non-infectious particles and DelVGs during single rounds of infection, shifting potential interactions among virions. Collectively, these findings open the possibility that the host cell’s metabolic signaling profile could directly modulate the infectivity of progeny Influenza A virions and the generation of DelVGs. Although much more research is needed, these results suggest that in principle, it would be possible to use metabolic signal modulation as a means to drive influenza infections toward milder clinical outcomes.

To our knowledge, our study is the first to examine the role of host metabolic state in the production of non-infectious influenza progeny particles and DelVGs [1, 20–22]. Specifically, we show differential dysregulation of PI3K signaling activity in a non-tumorigenic cell line (but see [61]) during infection by two different Influenza A strains, CA09 and TX12, then override this host-virus interaction using alpelisib, and finally show an increase in DelVGs and defective particle production. The host metabolic state can affect influenza infection in terms of clinical outcomes as shown in studies of obesity and cancer. For instance, obesity—a host metabolic state characterized by chronic inflammation and dysregulated immune responses—has been associated with increased titers of infectious progeny [32, 33]. Similarly, at the cellular level, cross-talk between Influenza A and host metabolic signaling effectors has been shown to affect the production of infectious progeny [39, 40, 64, 65]. However, the production of non-infectious progeny during different metabolic states had not previously been investigated; an extremely relevant line of investigation given that up to 90% of total viral particles are non-infectious [14–16], and that these particles have a role in clinical outcomes [1, 13]. Our findings confirm that interrupting virus-induced upregulation of host growth signaling can increase non-infectious Influenza A particle production, providing novel insight into the crosstalk between Influenza A and host metabolism. We highlight that this virus-host crosstalk is complex. For instance, we found a clear dose dependent effect of alpelisib on PI3K/pAKT in cells, as expected, because it is well documented that this is the molecular target of alpelisib. However, we did not have a particular expectation in the context of viral infection. PI3K functions as a master regulator of cellular metabolism, and its inhibition by alpelisib occurs within the broader context of multiple influenza virus–host interactions, including those mediated by the viral NS1 protein, which itself modulates PI3K activity. These intersecting influences could generate concentration-specific rather than linear dose-dependent effects on viral genome replication fidelity. We analyzed our data accordingly, with each concentration treated statistically as a different condition. Our goal was to show that Alpelisib could reproducibly show an effect on DelVGs and non-infectious particles, and not to investigate the mechanism. We acknowledge that the molecular mechanisms that underlie and influence non-infectious particle formation remain unclear in our study. Until now, investigations have focused primarily on the viral side of the equation, identifying viral genome mutations and infection multiplicity as variables influencing the production of non-infectious progeny across different strains [13, 25–27]. Our study’s contribution lies in demonstrating the possibility that metabolism can affect the production of non-infectious progeny and highlights the need to better understand the still unknown mechanisms to provide a more complete picture of how metabolic state affects Influenza A pathogenesis.

Our study combines single-cell immunofluorescence quantification with an automated assay that quantifies the proportion of non-infectious virus particles, providing a more accurate measurement of the infectious potential of a virus population [14]. Building on previous methods [14, 66, 67], our cluster-forming assay combined the infection localization of a conventional plaque assay with the immunocytochemical staining and microscopy of the standard immunofocus assay. By pairing this assay with an automated image analysis pipeline, we were able to capture influenza infectivity at a more detailed resolution than is possible with either parent assay alone (See Supplementary Material). By resolving NCUs (non-infectious particles) and PCUs (fully infectious particles) apart from each other, the cluster-forming assay has revealed strain-specific and dose-specific effects of alpelisib on key markers of defective interference at just 18 h.p.i. These novel outcomes represent early onset alterations to the trajectories of standard CA09 and TX12 infections, and each of these altered trajectories could potentially be desirable for different real-world therapeutic and prophylactic applications, provided they persist into later time points. Pharmacologically increasing the in situ spawn rate of non-infectious particles would potentially be a novel therapeutic possibility, provided the underlying physiological factors become better understood. A limitation of our study is the exact definition of the non-infectious particle component. Although the cluster-forming assay accurately titrates infectious and non-infectious particles, it was not designed to identify whether those non-infectious particles represent DIPs, particles with lethal or nonsense mutations, or particles that contain segments with defects in transcription [14]; an area that requires further study. We additionally expect the cluster-forming assay to facilitate future screens to uncover evermore druggable modulators of in situ non-infectious particles and DelVG production during Influenza A infection.

Based on the established cross-talk between effector proteins of both host cell metabolic signaling and Influenza A [39, 40, 64, 65], we hypothesized that pro-growth metabolic signal inhibition with alpelisib would induce abortive infectivity in progeny flu particles and increase DelVGs. Our predictions proved out, albeit in a strain-specific and concentration-specific manner. We speculate that the strain differences in PI3K activation we found (Fig. 2), potentially arising from differences in the NS1::p85β virus-host interaction (Fig. 1), and the specific metabolic state of the cell (suggested by the striking effects in the 20uM alpelisib concentration), will be crucial to changes in DelVG and non-infectious particle production. Uncovering more inducers that can have greater or more consistent effects will facilitate future investigations into the molecular mechanism through which DelVGs emerge and non-infectious progeny particles accumulate. Our results highlight the importance of host cell factors in determining the outcome of influenza virus infections, potentially informing host metabolic states that predict infection outcomes [68], as well as potential therapeutics that could induce host-mediated changes towards mild infection outcomes.

These findings suggest that the host context can shift the makeup of the DelVG population, shaping the potential virus-virus interactions [69–71] altering the course of the infection. Knowledge of these mechanisms will facilitate the development of more targeted abortive infectivity induction strategies for broad-spectrum anti-influenza therapeutics. DI is already being weaponized in the form of exogenously administered recombinant Influenza A virions called therapeutic interfering particles (TIPs), which have been engineered to contain one or more DelVGs. TIPs are propagation-incapable, and their administration attenuates Influenza A pathogenesis in a strain-indiscriminate [6, 10], dose-dependent manner [2–4, 6–10]. Our study opens the possibility of using host cell metabolic state as a strategic therapeutic target because of its readily responsive and reversible system-wide reach. Picomolar perturbations of host cell metabolism can drive system-wide reconfiguration of critical processes into countless unique endpoints; too many endpoints for Influenza A to possibly adapt against. Future research should address the composition of non-infectious progeny particles, and which PI3K downstream effector pathways transduce the signal(s) that ultimately impacts de novo non-infectious particle emergence. In sum, our research shows the promise of the host cell’s vast metabolic signaling network as a quick-response, therapeutically actionable, druggable target with the potential to steer flu pathology away from fatal towards mild outcomes.

## Supplementary Information

Additional file 1

## Data Availability

Data and analyses are posted on GitHub (https://github.com/pomoxis/alpelisib-SIP). Sequencing files will be deposited in the Sequencing Read Archive.

## References

[1] Dimmock NJ, Easton AJ. Defective interfering influenza virus RNAs: time to reevaluate their clinical potential as broad-spectrum antivirals? J Virol. 2014;88(10):5217–27. 10.1128/JVI.03193-13. (Epub 2014 Feb 26. PMID: 24574404; PMCID: PMC4019098). 24574404 10.1128/JVI.03193-13 PMC4019098

[2] Bdeir N, Arora P, Gärtner S, Hoffmann M, Reichl U, Pöhlmann S, et al. A system for production of defective interfering particles in the absence of infectious influenza A virus. PLoS ONE. 2019;14(3):e0212757. 10.1371/journal.pone.0212757. (PMID: 30822349; PMCID: PMC6396908). 30822349 10.1371/journal.pone.0212757 PMC6396908

[3] Harding AT, Haas GD, Chambers BS, Heaton NS. Influenza viruses that require 10 genomic segments as antiviral therapeutics. PLoS Pathog. 2019;15(11):e1008098. 10.1371/journal.ppat.1008098. (PMID: 31730644; PMCID: PMC6881065). 31730644 10.1371/journal.ppat.1008098 PMC6881065

[4] Meng B, Bentley K, Marriott AC, Scott PD, Dimmock NJ, Easton AJ. Unexpected complexity in the interference activity of a cloned influenza defective interfering RNA. Virol J. 2017;14(1):138. 10.1186/s12985-017-0805-6. (PMID: 28738877; PMCID: PMC5525295). 28738877 10.1186/s12985-017-0805-6 PMC5525295

[5] Pitchai FN, Tanner EJ, Khetan N, Vasen G, Levrel C, Kumar AJ, et al. Engineered deletions of HIV replicate conditionally to reduce disease in nonhuman primates. Science. 2024;385(9):eadn5866. 10.1126/science.adn5866. (PMID: 39116226 PMCID: PMC11545966). 39116226 10.1126/science.adn5866 PMC11545966

[6] Smith CM, Scott PD, O’Callaghan C, Easton AJ, Dimmock NJ. A defective interfering influenza RNA inhibits infectious influenza virus replication in human respiratory tract cells: a potential new human antiviral. Viruses. 2016;8(8):237. 10.3390/v8080237. (PMID:27556481;PMCID:PMC4997599). 27556481 10.3390/v8080237 PMC4997599

[7] Tapia F, Laske T, Wasik MA, Rammhold M, Genzel Y, Reichl U. Production of defective interfering particles of influenza A virus in parallel continuous cultures at two residence times-insights from qPCR measurements and viral dynamics modeling. Front Bioeng Biotechnol. 2019;7:275. 10.3389/fbioe.2019.00275. (PMID: 31681751; PMCID: PMC681321). 31681751 10.3389/fbioe.2019.00275 PMC6813217

[8] Wasik MA, Eichwald L, Genzel Y, Reichl U. Cell culture-based production of defective interfering particles for influenza antiviral therapy. Appl Microbiol Biotechnol. 2018;102(3):1167–77. 10.1007/s00253-017-8660-3. (Epub 2017 Dec 5. PMID: 29204901; PMCID: PMC5778153). 29204901 10.1007/s00253-017-8660-3 PMC5778153

[9] Yamagata Y, Muramoto Y, Miyamoto S, Shindo K, Nakano M, Noda T. Generation of a purely clonal defective interfering influenza virus. Microbiol Immunol. 2019;63(5):164–71. 10.1111/1348-0421.12681. (Epub 2019 May 17. PMID: 30997933). 30997933 10.1111/1348-0421.12681

[10] Zhao H, To KKW, Chu H, Ding Q, Zhao X, Li C, et al. Dual-functional peptide with defective interfering genes effectively protects mice against avian and seasonal influenza. Nat Commun. 2018;9(1):2358. 10.1038/s41467-018-04792-7. (PMID: 29907765; PMCID: PMC6004018). 29907765 10.1038/s41467-018-04792-7 PMC6004018

[11] Brennan JW, Sun Y. Defective viral genomes: advances in understanding their generation, function, and impact on infection outcomes. mBio. 2024;15(5):e0069224. 10.1128/mbio.00692-24. (Epub 2024 Apr 3. PMID: 38567955; PMCID: PMC11077978). 38567955 10.1128/mbio.00692-24 PMC11077978

[12] Felt SA, Sun Y, Jozwik A, Paras A, Habibi MS, Nickle D, et al. Detection of respiratory syncytial virus defective genomes in nasal secretions is associated with distinct clinical outcomes. Nat Microbiol. 2021;6(5):672–81. 10.1038/s41564-021-00882-3. (Epub 2021 Apr 1. PMID: 33795879; PMCID: PMC9098209). 33795879 10.1038/s41564-021-00882-3 PMC9098209

[13] Vasilijevic J, Zamarreño N, Oliveros JC, Rodriguez-Frandsen A, Gómez G, Rodriguez G, et al. Reduced accumulation of defective viral genomes contributes to severe outcome in influenza virus infected patients. PLoS Pathog. 2017;13(10):e1006650. 10.1371/journal.ppat.1006650. (PMID: 29023600; PMCID: PMC5638565). 29023600 10.1371/journal.ppat.1006650 PMC5638565

[14] Brooke CB, Ince WL, Wrammert J, Ahmed R, Wilson PC, Bennink JR, et al. Most influenza a virions fail to express at least one essential viral protein. J Virol. 2013;87(6):3155–62. 10.1128/JVI.02284-12. (PMID: 23283949; PMCID: PMC3592173). 23283949 10.1128/JVI.02284-12 PMC3592173

[15] Brooke CB. Population diversity and collective interactions during influenza virus infection. J Virol. 2017;91(27):e01164-17. 10.1128/JVI.01164-17. (PMID: 28855247; PMCID: PMC5660503). 28855247 10.1128/JVI.01164-17 PMC5660503

[16] Diefenbacher M, Sun J, Brooke CB. The parts are greater than the whole: the role of semi-infectious particles in influenza A virus biology. Curr Opin Virol. 2018;33:42–6. 10.1016/j.coviro.2018.07.002. (Epub 2018 Jul 24. PMID: 30053722; PMCID: PMC6642613). 30053722 10.1016/j.coviro.2018.07.002 PMC6642613

[17] Davis AR, Hiti AL, Nayak DP. Influenza defective interfering viral RNA is formed by internal deletion of genomic RNA. Proc Natl Acad Sci USA. 1980;77(1):215–9. 10.1073/pnas.77.1.215. (PMID: 6928614; PMCID: PMC348239). 6928614 10.1073/pnas.77.1.215 PMC348239

[18] Nayak DP, Sivasubramanian N, Davis AR, Cortini R, Sung J. Complete sequence analyses show that two defective interfering influenza viral RNAs contain a single internal deletion of a polymerase gene. Proc Natl Acad Sci U S A. 1982;79(7):2216–20. 10.1073/pnas.79.7.2216. (PMID: 6954536; PMCID: PMC346162). 6954536 10.1073/pnas.79.7.2216 PMC346162

[19] Saira K, Lin X, DePasse JV, Halpin R, Twaddle A, Stockwell T, et al. Sequence analysis of in vivo defective interfering-like RNA of influenza A H1N1 pandemic virus. J Virol. 2013;87(14):8064–74. 10.1128/JVI.00240-13. (Epub 2013 May 15. PMID: 23678180; PMCID: PMC3700204). 23678180 10.1128/JVI.00240-13 PMC3700204

[20] Manzoni TB, López CB. Defective (interfering) viral genomes re-explored: impact on antiviral immunity and virus persistence. Future Virol. 2018;13(7):493–503. 10.2217/fvl-2018-0021. (Epub 2018 Jun 12. PMID: 30245734; PMCID: PMC6136085). 30245734 10.2217/fvl-2018-0021 PMC6136085

[21] Vignuzzi M, López CB. Defective viral genomes are key drivers of the virus-host interaction. Nat Microbiol. 2019;4(7):1075–87. 10.1038/s41564-019-0465-y. (Epub 2019 Jun 3. PMID: 31160826; PMCID: PMC7097797). 31160826 10.1038/s41564-019-0465-y PMC7097797

[22] Wu M, Zhou E, Sheng R, Fu X, Li J, Jiang C, et al. Defective interfering particles of influenza virus and their characteristics, impacts, and use in vaccines and antiviral strategies: a systematic review. Viruses. 2022;14(12):2773. 10.3390/v14122773. (PMID:36560777;PMCID:PMC9781619). 36560777 10.3390/v14122773 PMC9781619

[23] Ranum JN, Ledwith MP, Alnaji FG, Diefenbacher M, Orton R, Sloan E, et al. Cryptic proteins translated from deletion-containing viral genomes dramatically expand the influenza virus proteome. Nucleic Acids Res. 2024;52(6):3199–212. 10.1093/nar/gkae133. (PMID:38407436;PMCID:PMC11014358). 38407436 10.1093/nar/gkae133 PMC11014358

[24] Alnaji FG, Holmes JR, Rendon G, Vera JC, Fields CJ, Martin BE, et al. Sequencing framework for the sensitive detection and precise mapping of defective interfering particle-associated deletions across influenza A and B viruses. J Virol. 2019;93(11):e00354-19. 10.1128/JVI.00354-19. (PMID: 30867305; PMCID: PMC6532088). 30867305 10.1128/JVI.00354-19 PMC6532088

[25] Von Magnus P. Incomplete forms of influenza virus. Adv Virus Res. 1954;2:59–79. 10.1016/s0065-3527(08)60529-1. (PMID: 13228257). 13228257 10.1016/s0065-3527(08)60529-1

[26] Rodriguez A, Falcon A, Cuevas MT, Pozo F, Guerra S, García-Barreno B, et al. Characterization *in vitro* and *in vivo* of a pandemic H1N1 influenza virus from a fatal case. PLoS ONE. 2013;8(1):e53515. 10.1371/journal.pone.0053515. (Epub 2013 Jan 10. PMID: 23326447; PMCID: PMC3542358). 23326447 10.1371/journal.pone.0053515 PMC3542358

[27] Perez-Cidoncha M, Killip MJ, Oliveros JC, Asensio VJ, Fernández Y, Bengoechea JA, et al. An unbiased genetic screen reveals the polygenic nature of the influenza virus anti-interferon response. J Virol. 2014;88(9):4632–46. 10.1128/JVI.00014-14. (Epub 2014 Feb 26. PMID: 24574395; PMCID: PMC3993829). 24574395 10.1128/JVI.00014-14 PMC3993829

[28] Alnaji FG, Brooke CB. Influenza virus DI particles: defective interfering or delightfully interesting? PLoS Pathog. 2020;16(5):e1008436. 10.1371/journal.ppat.1008436. (PMID: 32437428; PMCID: PMC7241698). 32437428 10.1371/journal.ppat.1008436 PMC7241698

[29] Winter G, Fields S, Ratti G. The structure of two subgenomic RNAs from human infhienza virus A/PR/8/34. Nucleic Acids Res. 1981;9(24):6907–15. 10.1093/nar/9.24.6907. (PMID: 7335495; PMCID: PMC327650). 7335495 10.1093/nar/9.24.6907 PMC327650

[30] Wang C, Honce R, Salvatore M, Chow D, Randazzo D, Yang J, et al. Influenza defective interfering virus promotes multiciliated cell differentiation and reduces the inflammatory response in mice. J Virol. 2023;97(6):e0049323. 10.1128/jvi.00493-23. (Epub 2023 May 31. PMID: 37255439; PMCID: PMC10308934). 37255439 10.1128/jvi.00493-23 PMC10308934

[31] Gardner EM, Beli E, Clinthorne JF, Duriancik DM. Energy intake and response to infection with influenza. Annu Rev Nutr. 2011;31(21):353–67. 10.1146/annurev-nutr-081810-160812. (PMID: 21548773). 21548773 10.1146/annurev-nutr-081810-160812

[32] Honce R, Schultz-Cherry S. Impact of obesity on influenza A virus pathogenesis, immune response, and evolution. Front Immunol. 2019;10:1071. 10.3389/fimmu.2019.01071. (PMID: 31134099; PMCID: PMC6523028). 31134099 10.3389/fimmu.2019.01071 PMC6523028

[33] Honce R, Karlsson EA, Wohlgemuth N, Estrada LD, Meliopoulos VA, Yao J, et al. Obesity-related microenvironment promotes emergence of virulent influenza virus strains. MBio. 2020;11(2):e03341-19. 10.1128/mBio.03341-19. (PMID: 32127459; PMCID: PMC7064783). 32127459 10.1128/mBio.03341-19 PMC7064783

[34] Ackermann WW, Klernschmidt E. Concerning the relation of the Krebs cycle to virus propagation. J Biol Chem. 1951;189(1):421–8 (PMID: 14832255). 14832255

[35] Lee SR, Roh JY, Ryu J, Shin HJ, Hong EJ. Activation of TCA cycle restrains virus-metabolic hijacking and viral replication in mouse hepatitis virus-infected cells. Cell Biosci. 2022;12(1):7 (PMCID: PMC8764321 PMID: 35042550). 35042550 10.1186/s13578-021-00740-z PMC8764321

[36] Ohno M, Sekiya T, Nomura N, Daito TJ, Shingai M, Kida H. Influenza virus infection affects insulin signaling, fatty acid-metabolizing enzyme expressions, and the tricarboxylic acid cycle in mice. Sci Rep. 2020;10(1):10879. 10.1038/s41598-020-67879-6. (PMID:32616893;PMCID:PMC7331672). 32616893 10.1038/s41598-020-67879-6 PMC7331672

[37] Hopkins BD, Goncalves MD, Cantley LC. Insulin-PI3K signalling: an evolutionarily insulated metabolic driver of cancer. Nat Rev Endocrinol. 2020;16(5):276–83. 10.1038/s41574-020-0329-9. (Epub 2020 Mar 3. PMID: 32127696; PMCID: PMC7286536). 32127696 10.1038/s41574-020-0329-9 PMC7286536

[38] Wee P, Wang Z. Epidermal growth factor receptor cell proliferation signaling pathways. Cancers (Basel). 2017;9(5):52. 10.3390/cancers9050052. (PMID: 28513565; PMCID: PMC5447962). 28513565 10.3390/cancers9050052 PMC5447962

[39] Hale BG, Jackson D, Chen YH, Lamb RA, Randall RE. Influenza A virus NS1 protein binds p85β and activates phosphatidylinositol-3-kinase signaling. Proc Natl Acad Sci U S A. 2006;103:14194–9. 10.1073/pnas.0606109103. (Epub 2006 Sep 8. PMID: 16963558; PMCID: PMC1599933). 16963558 10.1073/pnas.0606109103 PMC1599933

[40] Li Y, Anderson DH, Liu Q, Zhou Y. Mechanism of influenza A virus NS1 protein interaction with the p85beta, but not the p85alpha, subunit of phosphatidylinositol 3-kinase (PI3K) and up-regulation of PI3K activity. J Biol Chem. 2008;283(34):23397–409. 10.1074/jbc.M802737200. (Epub 2008 Jun 5 PMID: 18534979). 18534979 10.1074/jbc.M802737200

[41] Al-Saffar NM, Jackson LE, Raynaud FI, Clarke PA, Ramírez de Molina A, Lacal JC, Workman P, Leach MO. The phosphoinositide 3-kinase inhibitor PI-103 downregulates choline kinase alpha leading to phosphocholine and total choline decrease detected by magnetic resonance spectroscopy. Cancer Res. 2010;70(13):5507–17. 10.1158/0008-5472.CAN-09-4476. Epub 2010 Jun 15. PMID: 20551061; PMCID: PMC2896552. 10.1158/0008-5472.CAN-09-4476 PMC2896552 20551061

[42] Luo Y, Xu W, Li G, Cui W. Weighing in on mTOR complex 2 signaling: the expanding role in cell metabolism. Oxid Med Cell Longev. 2018;2018:7838647. 10.1155/2018/7838647. (PMID: 30510625; PMCID: PMC6232796). 30510625 10.1155/2018/7838647 PMC6232796

[43] Saha A, Connelly S, Jiang J, Zhuang S, Amador DT, Phan T, et al. Akt phosphorylation and regulation of transketolase is a nodal point for amino acid control of purine synthesis. Mol Cell. 2014;55(2):264–76. 10.1016/j.molcel.2014.05.028. (Epub 2014 Jun 26. PMID: 24981175; PMCID: PMC4104231). 24981175 10.1016/j.molcel.2014.05.028 PMC4104231

[44] Furet P, Guagnano V, Fairhurst RA, Imbach-Weese P, Bruce I, Knapp M, et al. Discovery of NVP-BYL719 a potent and selective phosphatidylinositol-3 kinase alpha inhibitor selected for clinical evaluation. Bioorg Med Chem Lett. 2013;23(13):3741–8. 10.1016/j.bmcl.2013.05.007. (Epub 2013 May 14. Erratum in: Bioorg Med Chem Lett. 2013 Aug 15;23(16):4723. PMID: 23726034). 23726034 10.1016/j.bmcl.2013.05.007

[45] Fritsch C, Huang A, Chatenay-Rivauday C, Schnell C, Reddy A, Liu M, et al. Characterization of the novel and specific PI3Kα inhibitor NVP-BYL719 and development of the patient stratification strategy for clinical trials. Mol Cancer Ther. 2014;13(5):1117–29. 10.1158/1535-7163.MCT-13-0865. (Epub 2014 Mar 7. PMID: 24608574). 24608574 10.1158/1535-7163.MCT-13-0865

[46] Yang J, Nie J, Ma X, Wei Y, Peng Y, Wei X. Targeting PI3K in cancer: mechanisms and advances in clinical trials. Mol Cancer. 2019;18(1):26. 10.1186/s12943-019-0954-x. (PMID: 30782187; PMCID: PMC6379961). 30782187 10.1186/s12943-019-0954-x PMC6379961

[47] Pargett M, Gillies TE, Teragawa CK, Sparta B, Albeck JG. Single-Cell Imaging of ERK Signaling Using Fluorescent Biosensors. Methods Mol Biol. 2017;1636:35–59. 10.1007/978-1-4939-7154-1_3. (PMID:28730471;PMCID:PMC8005261). 28730471 10.1007/978-1-4939-7154-1_3 PMC8005261

[48] Baker SF, Guo H, Albrecht RA, García-Sastre A, Topham DJ, Martínez-Sobrido L. Protection against lethal influenza with a viral mimic. J Virol. 2013;87(15):8591–605. 10.1128/JVI.01081-13. (Epub 2013 May 29. PMID: 23720727; PMCID: PMC3719819). 23720727 10.1128/JVI.01081-13 PMC3719819

[49] Jaworski E, Routh A. Parallel ClickSeq and Nanopore sequencing elucidates the rapid evolution of defective-interfering RNAs in Flock House virus. PLoS Pathog. 2017;13(5):e1006365. 10.1371/journal.ppat.1006365. (PMID: 28475646; PMCID: PMC5435362). 28475646 10.1371/journal.ppat.1006365 PMC5435362

[50] Routh A, Johnson JE. Discovery of functional genomic motifs in viruses with ViReMa-a virus recombination mapper-for analysis of next-generation sequencing data. Nucleic Acids Res. 2014;42(2):e11. 10.1093/nar/gkt916. (Epub 2013 Oct 16. PMID: 24137010; PMCID: PMC3902915). 24137010 10.1093/nar/gkt916 PMC3902915

[51] Sotcheff S, Zhou Y, Yeung J, Sun Y, Johnson JE, Torbett BE, et al. Virema: a virus recombination mapper of next-generation sequencing data characterizes diverse recombinant viral nucleic acids. Gigascience. 2023;12:giad009. 10.1093/gigascience/giad009. (PMID: 36939008; PMCID: PMC10025937). 36939008 10.1093/gigascience/giad009 PMC10025937

[52] Russell AB, Trapnell C, Bloom JD. Extreme heterogeneity of influenza virus infection in single cells. Elife. 2018;7:e32303. 10.7554/eLife.32303. (PMID: 29451492; PMCID: PMC5826275). 29451492 10.7554/eLife.32303 PMC5826275

[53] Wang C, Forst CV, Chou TW, Geber A, Wang M, Hamou W, et al. Cell-to-cell variation in defective virus expression and effects on host responses during influenza virus infection. MBio. 2020;11(1):e02880-19. 10.1128/mBio.02880-19. (PMID: 31937643; PMCID: PMC6960286). 31937643 10.1128/mBio.02880-19 PMC6960286

[54] Lopes AM, Domingues P, Zell R, Hale BG. Structure-guided functional annotation of the Influenza A virus NS1 protein reveals dynamic evolution of the p85β-binding site during circulation in humans. J Virol. 2017;91(21):e01081-17. 10.1128/JVI.01081-17. (PMID: 28814525; PMCID: PMC564087). 28814525 10.1128/JVI.01081-17 PMC5640874

[55] Cho JH, Zhao B, Shi J, Savage N, Shen Q, Byrnes J, et al. Molecular recognition of a host protein by NS1 of pandemic and seasonal influenza A viruses. Proc Natl Acad Sci U S A. 2020;117(12):6550–8. 10.1073/pnas.1920582117. (Epub 2020 Mar 9. PMID: 32152123; PMCID: PMC7104383). 32152123 10.1073/pnas.1920582117 PMC7104383

[56] Ayllon J, Hale BG, García-Sastre A. Strain-specific contribution of NS1-activated phosphoinositide 3-kinase signaling to influenza A virus replication and virulence. J Virol. 2012;86(9):5366–70. 10.1128/JVI.06722-11. (PMID: 22345452; PMCID: PMC3347353). 22345452 10.1128/JVI.06722-11 PMC3347353

[57] Fruman DA, Rommel C. PI3K and cancer: lessons, challenges and opportunities. Nat Rev Drug Discov. 2014;13(2):140–56. 10.1038/nrd4204. (PMID: 24481312; PMCID: PMC3994981). 24481312 10.1038/nrd4204 PMC3994981

[58] Fruman DA, Chiu H, Hopkins BD, Bagrodia S, Cantley LC, Abraham RT. The PI3K pathway in human disease. Cell. 2017;170(4):605–35. 10.1016/j.cell.2017.07.029. (PMID:28802037;PMCID:PMC5726441). 28802037 10.1016/j.cell.2017.07.029 PMC5726441

[59] Jokinen E, Koivunen JP. MEK and PI3K inhibition in solid tumors: rationale and evidence to date. Ther Adv Med Oncol. 2015;7(3):170–80. 10.1177/1758834015571111. 26673580 10.1177/1758834015571111 PMC4406912

[60] Yuan TL, Cantley LC. PI3K pathway alterations in cancer: variations on a theme. Oncogene. 2008;27(18):5497–510. 10.1038/onc.2008.245. (PMID: 18794884; PMCID: PMC3398461). 18794884 10.1038/onc.2008.245 PMC3398461

[61] Yang D, Huang L, Wang J, Wu H, Liu Z, Abudureyimu A, et al. Tumorigenesis mechanism and application strategy of the MDCK cell line: a systematic review. Biologicals. 2023;83:101699. 10.1016/j.biologicals.2023.101699. (Epub 2023 Aug 11. PMID: 37573790). 37573790 10.1016/j.biologicals.2023.101699

[62] Cooper PD. The plaque assay of animal viruses. Adv Virus Res. 1961;8:319–78. 10.1016/s0065-3527(08)60689-2. (PMID: 13881155). 13881155 10.1016/s0065-3527(08)60689-2

[63] Matrosovich M, Matrosovich T, Garten W, Klenk HD. New low-viscosity overlay medium for viral plaque assays. Virol J. 2006;31(3):63. 10.1186/1743-422X-3-63. (PMID:16945126;PMCID:PMC1564390). 10.1186/1743-422X-3-63 PMC1564390 16945126

[64] Kuss-Duerkop SK, Wang J, Mena I, White K, Metreveli G, Sakthivel R, et al. Influenza virus differentially activates mTORC1 and mTORC2 signaling to maximize late stage replication. PLoS Pathog. 2017;13(9):e1006635. 10.1371/journal.ppat.1006635. (PMID:28953980;PMCID:PMC5617226). 28953980 10.1371/journal.ppat.1006635 PMC5617226

[65] Smallwood HS, Duan S, Morfouace M, Rezinciuc S, Shulkin BL, Shelat A, et al. Targeting metabolic reprogramming by influenza infection for therapeutic intervention. Cell Rep. 2017;19(23):1640–53. 10.1016/j.celrep.2017.04.039. (PMID: 28538182; PMCID: PMC5599215). 28538182 10.1016/j.celrep.2017.04.039 PMC5599215

[66] Amarilla AA, Modhiran N, Setoh YX, Peng NYG, Sng JDJ, Liang B, et al. An Optimized High-Throughput Immuno-Plaque Assay for SARS-CoV-2. Front Microbiol. 2021;12(12):625136. 10.3389/fmicb.2021.625136. (PMID:33643253;PMCID:PMC7906992). 33643253 10.3389/fmicb.2021.625136 PMC7906992

[67] Cacciabue M, Currá A, Gismondi MI. Viralplaque: a Fiji macro for automated assessment of viral plaque statistics. PeerJ. 2019;7:e7729. 10.7717/peerj.7729. (PMID: 31579606; PMCID: PMC6764358). 31579606 10.7717/peerj.7729 PMC6764358

[68] Engels G, Hierweger AM, Hoffmann J, Thieme R, Thiele S, Bertram S, et al. Pregnancy-related immune adaptation promotes the emergence of highly virulent H1N1 influenza virus strains in allogenically pregnant mice. Cell Host Microbe. 2017;21(8):321–33. 10.1016/j.chom.2017.02.020. (PMID: 28279344). 28279344 10.1016/j.chom.2017.02.020

[69] Alnaji FG, Farjo M, Goh SL, Hao Aw DZ, Lorenzini PA, Liu T, Lin-Zhen RC, Lee C, Vignuzzi M, Brooke CB. Longitudinal analysis of influenza A virus deletion-containing viral genomes reveals key determinants of co-evolutionary dynamics and interference. bioRxiv [Preprint]. 2025:2025.11.03.686333. 10.1101/2025.11.03.686333. PMID: 41279761; PMCID: PMC12637726. 10.1038/s41467-026-74526-7 PMC13522585 42303613

[70] Díaz-Muñoz SL, Sanjuán R, West S. Sociovirology: conflict, cooperation, and communication in viruses. Cell Host Microbe. 2017;22(4):437–41 (PMID:29024640). 29024640 10.1016/j.chom.2017.09.012 PMC5644717

[71] Leeks A, Bono LM, Ampolini EA, Souza LS, Höfler T, Mattson CL, et al. Open questions in the social lives of viruses. J Evol Biol. 2023;36:1551–67 (PMID: 37975507). 37975507 10.1111/jeb.14203 PMC11281779

