# Supplementary material for: Host PI3K inhibition via anti-cancer drug alpelisib influences Influenza A non-infectious particles and deletion-containing viral genomes
Source: Cell Commun Signal. 2025 Dec 19;23:543. doi: 10.1186/s12964-025-02598-x (PMC12751567; doi:10.1186/s12964-025-02598-x)
Supplement: Supplementary file 1 — Additional file 1 [file 12964_2025_2598_MOESM1_ESM.pdf]

## SUPPLEMENTARY MATERIALS

# Host PI3K inhibition via anti-cancer drug alpelisib influences Influenza A non-infectious particles and deletion-containing viral genomes

Ilechukwu Agu[1], Ivy José[1], Abhineet Ram[2], Daniel Oberbauer[2], John Albeck[2], Samuel L. Díaz Muñoz[1, 3*]

## Extended Results

### *Alpelisib inhibits PI3K network signaling in MDCK-London cells*

We set out to confirm that alpelisib inhibits PI3K network signaling in the MDCK-London cell line, as has been demonstrated in numerous other mammalian cell lines and cell-free assays (Furet, 2013; Fritsch, 2014; Yang, 2019). We leveraged immunocytochemistry and fluorescence microscopy to quantify pAKT activity at the single cell level (Pargett, 2017). We found strong evidence that alpelisib inhibited PI3K network signaling by measuring the activity of the downstream pAKT effector. Increased doses of alpelisib, across a broad 1.25 - 40 µM concentration range, resulted in a clear dose dependent decrease in pAKT activity (Adjusted $R^2 = 0.1672$, $p < 0.00001$, **Figure 1A**), with a 4.5342 decrease in AU per µM of alpelisib. In qualitative terms, pAKT activity was clearly downregulated in the alpelisib-treated monolayer (**Figure 1B**) relative to the vehicle-treated monolayer (**Figure 1C**); whose baseline pAKT activity was dusted across the cytoplasm of some cells and focused in the nuclei of others. Expectedly, the insulin-treated positive control group showed vivid pAKT upregulation, wherein pAKT activity localization went past cytoplasm and nuclei to include the plasma membrane (**Figure 1D**, **Supplementary Figure S1**).

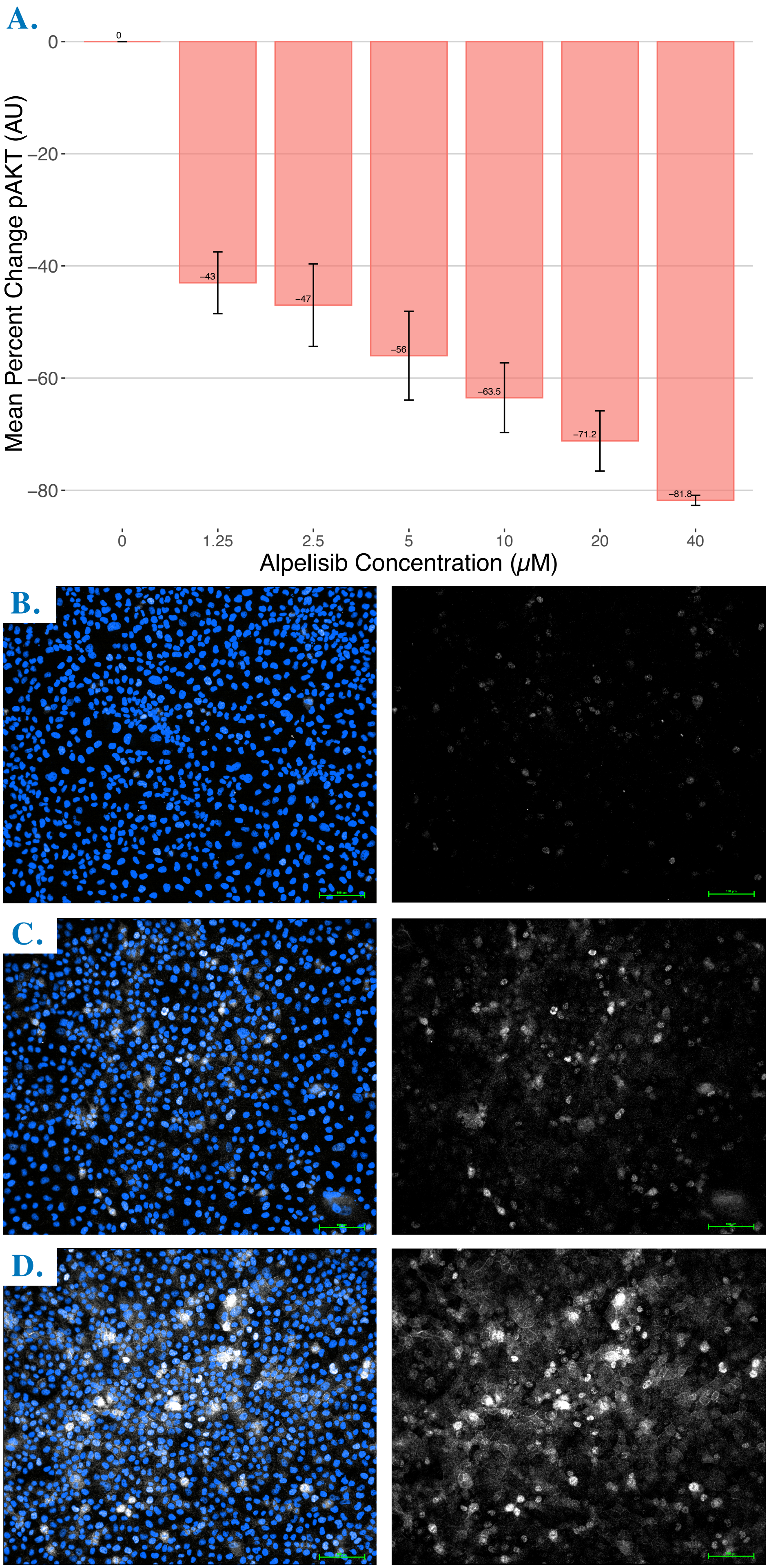
A.
B.
C.
D.
Mean Percent Change pAKT (AU)
0
−20
−40
−60
−80
Alpelisib Concentration (μM)
0
1.25
2.5
5
10
20
40
0
−43
−47
−56
−63.5
−71.2
−81.8

**Supplementary Figure S1. Alpelisib decreases pAKT activity (AU) of MDCK-London in a dose dependent manner.** (**A**) Mean percent change in pAKT activity (AU) of MDCK-London cells exposed to increasing concentrations of alpelisib. Bars represent the average of three biological replicates and error bars depict the SEM. 0 µM alpelisib treatment group received vehicle solvent (DMSO) . Fluorescence microscopy images of MDCK monolayers (**B-D**). (**B**) 20 µM alpelisib treated monolayer, (**C**) Vehicle treated monolayer, (**D**) Insulin treated monolayer (positive control). White/Cy5 – pAKT(S473); Blue/Hoechst – MDCK-London nucleus; green scale bar = 100µM.

## *Insulin activates PI3K network signaling in MDCK-London cells*

In addition to exposing uninfected and virus-infected MDCK-London cells to increasing concentrations of alpelisib, a parallel experiment was run in which the same test groups were co-exposed to 10ug/mL of insulin. This experiment confirmed the potentiating effect of insulin on PI3K-AKT signaling activation (positive control for pAKT activation). pAKT activation levels in all parallel insulin exposed experiment groups are higher than their no-insulin counterparts (**Figure S2**).

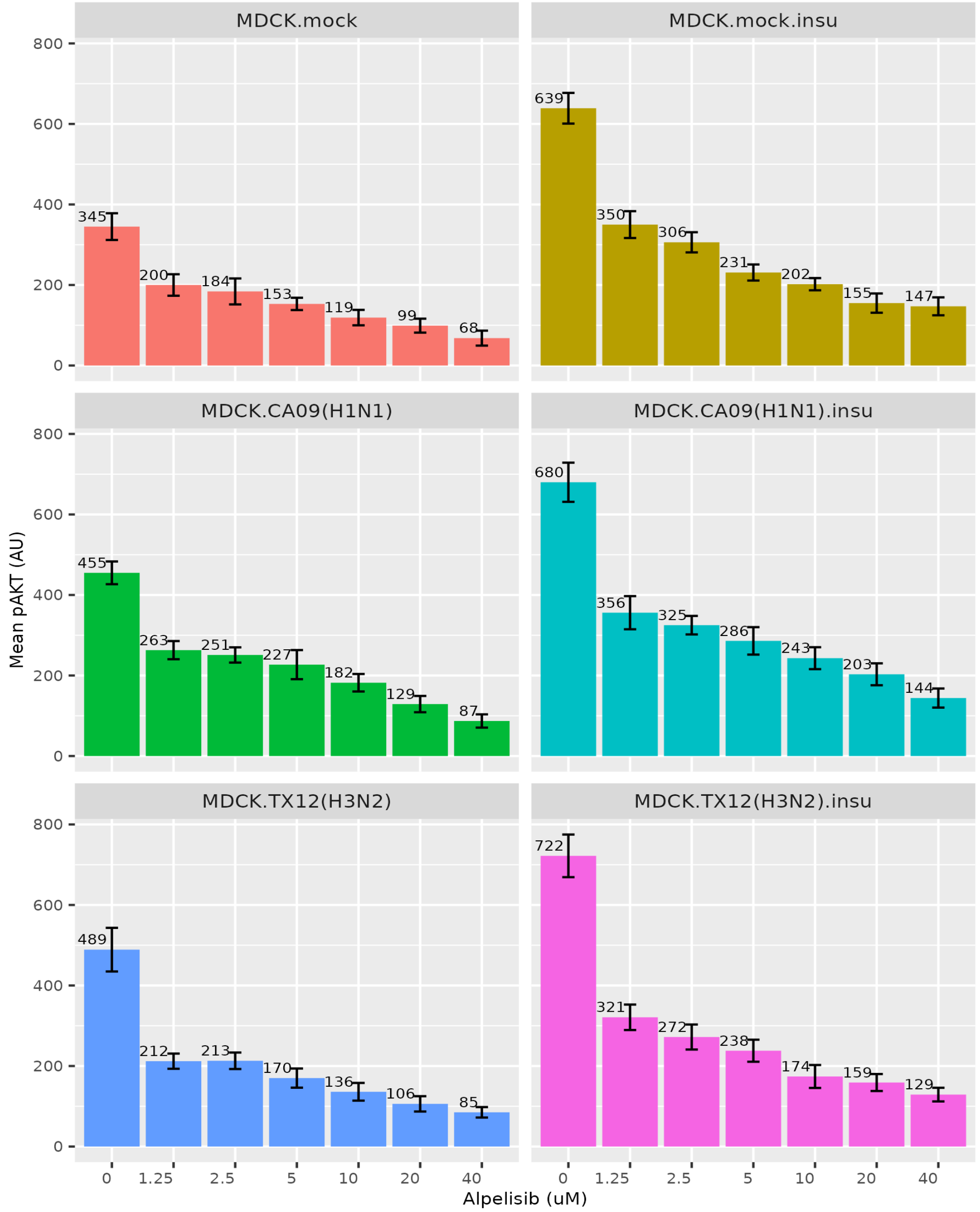


**Supplementary Figure S2.** pAKT activation in mock- and virus-infected MDCK-London cells that were either exposed to different concentrations of alpelisib (left), or co-exposed to 10ug/mL of insulin in addition to

alpelisib (right). 0 µM alpelisib treatment group received vehicle solvent (DMSO). Bars represent the average of three biological replicates and error bars depict the SEM.

## *Alpelisib affects production of deletion-containing viral genomes early in Influenza A infection*

Our statistical model allowed us to estimate an effect for each segment and alpelisib treatment (**Table S1**) on DelVGs and TVGs.

| Strain | Measure | Segment | Alpelisib Treatment (µM) | Estimate | Std. Error | t value | Pr(>\|t\|) | |
|---|---|---|---|---|---|---|---|---|
| **CA09** | PropDVG | PB2 | 20 | 0.2473223 | 0.0679473 | 3.64 | 0.00339 | ** |
| **CA09** | PropDVG | PB1 | 20 | 0.373685 | 0.102129 | 3.659 | 0.00327 | ** |
| **CA09** | PropDVG | HA | 20 | 0.0578874 | 0.0168425 | 3.437 | 0.00492 | ** |
| **TX12** | PropDVG | HA | 1.25 | -0.0060637 | 0.0008546 | -7.095 | 1.26E-05 | *** |
| **TX12** | PropDVG | HA | 2.5 | -0.0024982 | 0.0008546 | -2.923 | 1.28E-02 | * |
| **TX12** | PropDVG | HA | 5 | -0.0043145 | 0.0008546 | -5.048 | 0.000285 | *** |
| **TX12** | PropDVG | HA | 10 | -0.0026667 | 0.0008546 | -3.12 | 0.00885 | ** |
| **TX12** | PropDVG | M | 1.25 | -0.0048573 | 0.0011397 | -4.262 | 0.0011 | ** |
| **CA09** | TVG | PB2 | 20 | -5173.3 | 1893.1 | -2.733 | 1.82E-02 | * |
| **CA09** | TVG | PB1 | 20 | -2371 | 1040.4 | -2.279 | 4.18E-02 | * |
| **CA09** | TVG | PA | 20 | -6456 | 2235 | -2.889 | 1.36E-02 | * |
| **CA09** | TVG | HA | 20 | -6840 | 2576 | -2.655 | 0.02099 | * |
| **CA09** | TVG | NA | 20 | -6758 | 2921 | -2.314 | 0.039197 | * |

**Supplementary Table S1. Alpelisib affects the proportion of deletion containing viral genomes and total viral genomes produced in infections**. Statistically significant predictors of the average size of of **Del**etion-containing **V**iral **G**enomes (DelVGs, blue shading) and and Total Viral Genomes (TVG, gray shading) including their parameter estimates from per-segment linear mixed models.

## *Alpelisib increases average deletion size in the polymerase segments*

We found Alpelisib increased the number of DelVGs, and wanted to examine how many different DelVGs were formed and distribution of DelVGs also changed. To meet this goal, we used ViReMa to enumerate all the unique species of DelVGs in each segment and used linear mixed models to test for differences among alpelisib concentrations; that is we examined how many different DelVGs were formed, but did not evaluate their abundance in these analyses. Alpelisib increased the average deletion size of polymerase complex segment DelVGs species in both the H1N1 and H3N2 strain (**Figure S4**, **Table S2**). Specifically, CA09 infections with 20µM of alpelisib had increases in average deletion size on all three polymerase segments (PB2: 124.00 bps; PB1: 193.78 bps; PA: 148.511 bps) and the HA segment (52.846 bps). TX12 registered more modest increases in average deletion size in the PB2 (20µM: 61.96 bps) and PB1 (20µM:_73.45 bps; 40µM: 78.46) segments. Additionally, CA09 also registered *decreases* in average PB2 deletion sizes ranging from 64.287 - 85.754 bps at the 1.25, 2.5, and 5µM concentrations. The change in the average deletion size in the DelVG population appeared to be due to an overall decrease of DelVGs with small deletions and most of the remaining DelVGs having larger deletions (**Figure S4**).

To compare the DelVG composition to previously published results, we constructed parallel coordinate plots, as in Alnaji et al. (2019) (**Figure S5**). Alnaji et al. (2019) labeled their strain Cal07, a reverse genetics rescue of strain A/California/04/2009 with mutations in HA and NP to match the sequence of the same strain we use here (A/California/07/2009). Alnaji et al. (2019) present the specific location of deletion junctions of Cal07 PB1 and PA after *six* high MOI passages which promote DelVG formation and persistence. Our plots at 20µM alpelisib look qualitatively similar, a fact that is notable, as we only minimized multiple cycles of replication and used a

low MOI. Given this difference in methodology, the similarity in PB1 (in particular) between the different studies is striking, suggesting that an infection with alpelisib at the 20μM concentration generates a DelVG distribution similar to multiple high MOI passages. As with the DelVG abundance data, which showed no statistical differences in DelVG abundance at concentrations other than 20μM, the other concentrations do not show qualitative differences in junctions to the 0μM treatment, so the corresponding parallel coordinate plots are not shown.

| Strain | Measure | Segment | Alpelisib Treatment (μM) | Estimate | Std. Error | t value | Pr(>\|t\|) | |
|---|---|---|---|---|---|---|---|---|
| **CA09** | size (bp) | PB2 | 1.25 | -69.648 | 28.061 | -2.482 | 1.31E-02 | * |
| **CA09** | size (bp) | PB2 | 2.5 | -85.754 | 25.689 | -3.338 | 8.49E-04 | *** |
| **CA09** | size (bp) | PB2 | 5 | -64.287 | 27.952 | -2.3 | 2.15E-02 | * |
| **CA09** | size (bp) | PB2 | 20 | 124.005 | 31.561 | 3.929 | 8.63E-05 | *** |
| **TX12** | size (bp) | PB2 | 20 | 61.96 | 30.2 | 2.052 | 0.0402 | * |
| **CA09** | size (bp) | PB1 | 20 | 193.782 | 49.145 | 3.943 | 8.23E-05 | *** |
| **TX12** | size (bp) | PB1 | 20 | 73.45 | 32.35 | 2.27 | 0.0232 | * |
| **TX12** | size (bp) | PB1 | 40 | 78.46 | 32.3 | 2.429 | 0.0152 | * |
| **CA09** | size (bp) | PA | 20 | 148.511 | 23.739 | 6.256 | 4.15E-10 | *** |
| **CA09** | size (bp) | HA | 20 | 52.846 | 18.431 | 2.867 | 4.16E-03 | ** |

Supplementary Table S2. **Alpelisib affects average deletion size in infections**. Statistically significant predictors of the average size of **Del**etion-containing **V**iral **G**enomes and their parameter estimates from per-segment linear mixed models.

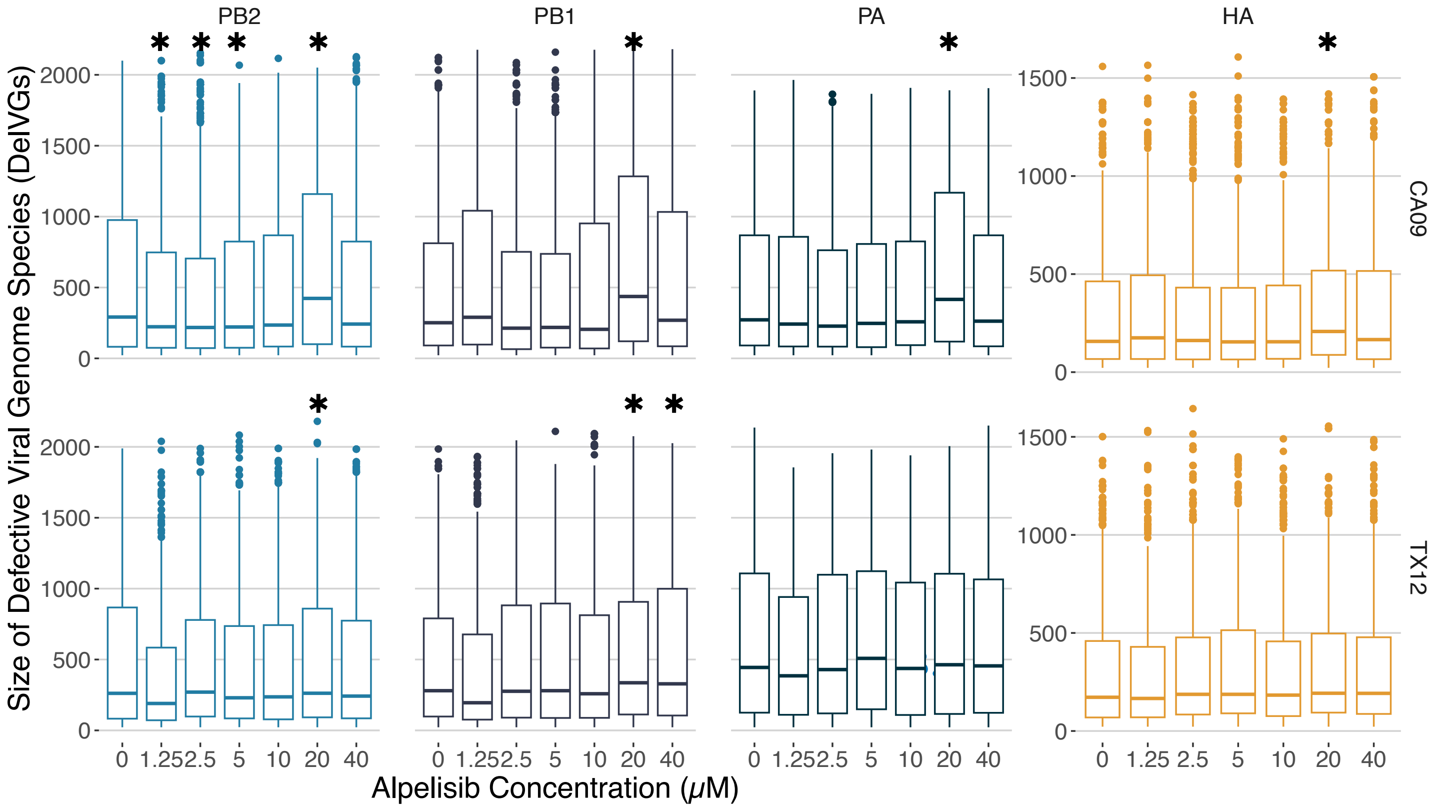


Supplementary Figure S4**. Alpelisib increases average deletion size in the polymerase segments.** Size (in basepairs, bps) distribution of distinct deletions in defective viral genomes (here measured **Del**etion-containing **V**iral **G**enomes (DelVGs) produced by CA09 (H1N1) and TX12 (H3N2) infections under different

concentrations of Alpelisib. Asterisks indicate statistically significant changes in mean size compared to the mock infection. Top row: CA09 (H1N1) strain; bottom row: TX12 (H3N2) strain. Boxplot colors represent different genome segments, note the different y-axis scale in segment HA plot.

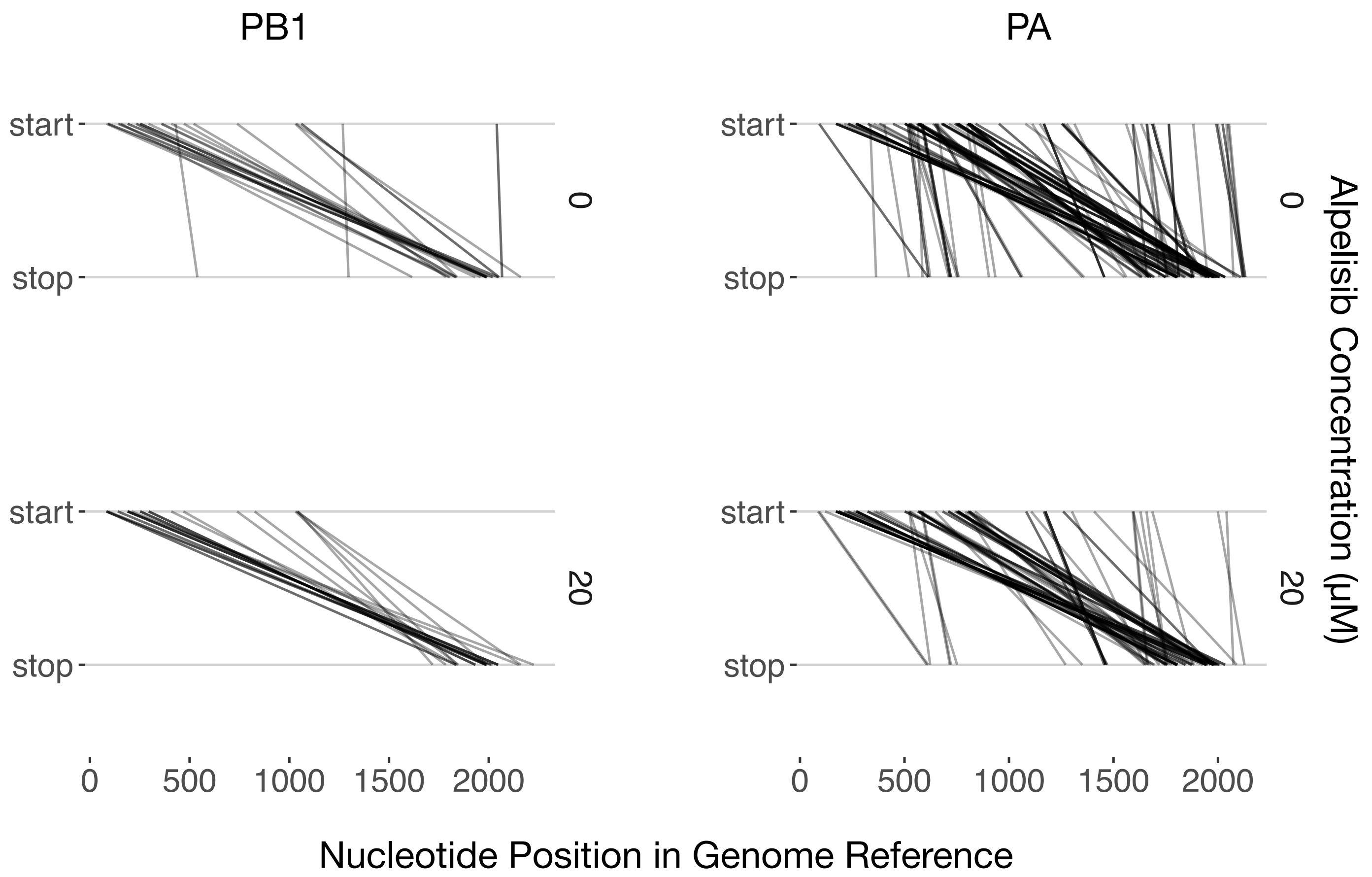


**Supplementary Figure S5.** Parallel coordinates plot depicting start and stop nucleotide positions of PB1 and PA DelVGs found in the CA09 strain, at different alpelisib concentrations (0µM, top; 20µM, bottom). Each line represents a recombination event that resulted in a deletion, plot only includes those unique junctions (same start and stop) found in all three bioreplicates. Only PA and PB1 data are shown to compare to published results on a similar strain (Cal07) in Figure 9C of Alnaji et al. 2019.

## Extended Methods

### *Cluster-forming Assay*

The cluster-forming assay combines the infection localization of a conventional plaque assay with the immunofluorescence (IF) staining and microscopy of a conventional immunofocus assay to capture influenza infectivity at a deeper resolution than possible with either parent assay alone.

Overnight MDCK-London cells—1.04 x $10^5$ total cells per well—were seeded into collagen-treated, glass-bottom 96-well tissue culture plates in MEM plus 5% FBS media to achieve 100% confluence in 24 hr. Confluent monolayers were then inoculated with serial dilutions of virus stock and incubated for 1 hr to allow for virus-monolayer adsorption, after which inoculum was aspirated and monolayers washed with MEM plus 2% bovine serum albumin and 1% Anti-Anti (Virus Infection Media; VIM). At this juncture, the conventional plaque assay or immunofocus assay would respectively see a solid or liquid overlay medium applied to the inoculated monolayers. The cluster-forming assay, on the other hand, applies a low to medium-viscosity overlay medium (VIM plus 4% carboxymethyl cellulose and 1 ug/mL TPCK-Trypsin) that remains in a semi-solid state at the end of the assay. This viscous overlay restricts diffusion of progeny virus to directly adjacent cells much like a conventional plaque assay, but has the added benefit of being removable via an aspirator pipette so that monolayers may be fixed, stained with IF antibodies, and imaged. Overlaid monolayers were incubated an additional 11 hours, at which point overlay media was aspirated and monolayers fixed with 4% PFA. Fixed monolayers were stained with fluorophore-conjugated IF antibodies targeting Influenza A nucleoprotein, counterstained with Hoechst, and imaged via fluorescence microscopy. The output at this juncture is an IF image of an flu-infected monolayer (**Supplementary Figure 3.1**) wherein each cluster of infected cells represents a productive infection mounted by a single propagation-capable virion—a *productive clustering unit* (PCU)—while solitary infection foci represent abortive infections mounted by propagation-incapable virus—*non-clustering units* (NCU).

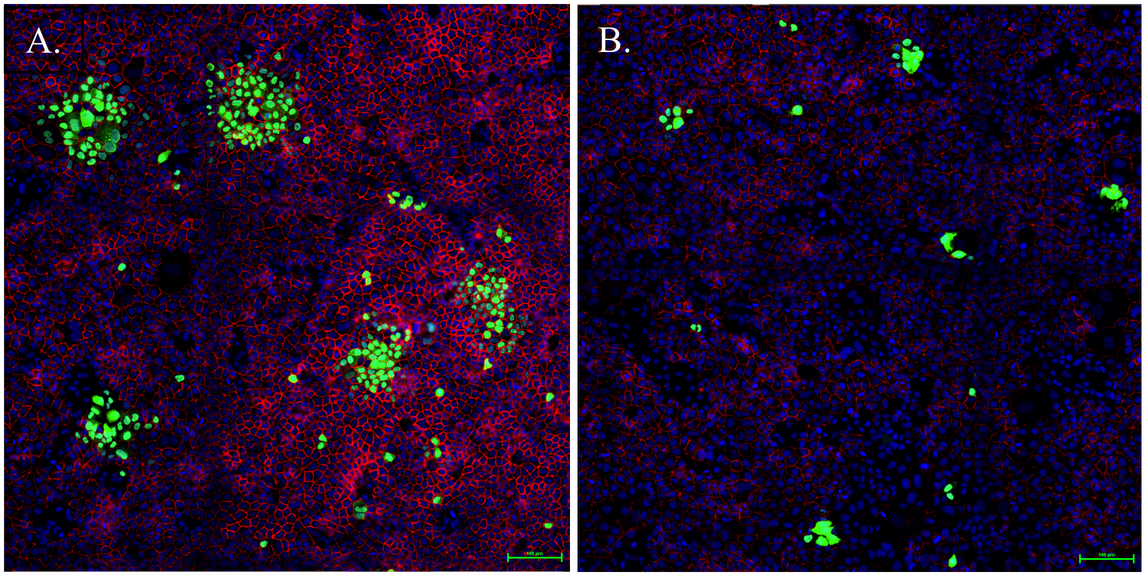


**Supplementary Figure S3.1**. Cluster-forming assay of Influenza A Virus on MDCK-London cells showing productive and abortive infections. Green/GFP – A/California/07/2009 nucleoprotein (**A**), A/Texas/50/2012 nucleoprotein (**B**); Blue/Hoechst – MDCK-London nucleus; Red/Cy5 – MDCK-London E-cadherin (**A**) or β-catenin (**B**).

Our proof of concept cluster-forming assays worked as designed; propagation-incapable virus mounted abortive infections as evidenced by NCUs, while propagation-capable virus mounted productive infections as evidenced by PCUs. Ordinarily, PCUs and NCUs would be tallied and titrated, but minor optimization of a few parameters was necessary to boost assay precision. Chief among these parameters were *monolayer integrity* and *PCU spillover*.

**Monolayer Integrity**: Monolayer damage and stripping undermines cluster-forming assay precision because the signal of an infection event is diminished—or lost outright—with each sloughed cell. This is especially relevant for abortive infections, whose assay signal is transmitted by a single cell, the sloughing of which results in underreporting and underestimation of NCU titer and NCU proportion. Therefore, it is imperative that monolayer integrity be preserved at all steps. We discovered that not prewarming reagents to room temperature or 37 °C—i.e. rapid reduction in temperature—caused monolayers to peel (**Supplementary Figure 3.2A**). That said, monolayer damage was consistently and primarily observed in monolayer regions of lower cell density (**Supplementary Figure 3.2B**). We found that seeding at higher cell density—upwards of $1.6 \times 10^4$ to $1.04 \times 10^5$ cells per well—and seeding more evenly with a wide-bore 2mL serological pipette in place of a multichannel pipette was sufficient to mitigate monolayer damage in future assays (**Supplementary Figure 3.3A**). However, PCU spillover and streaks remained an issue; especially for a fast growing strain like the pandemic A/California/07/2009(H1N1) (**Supplementary Figure 3.3B-C**).

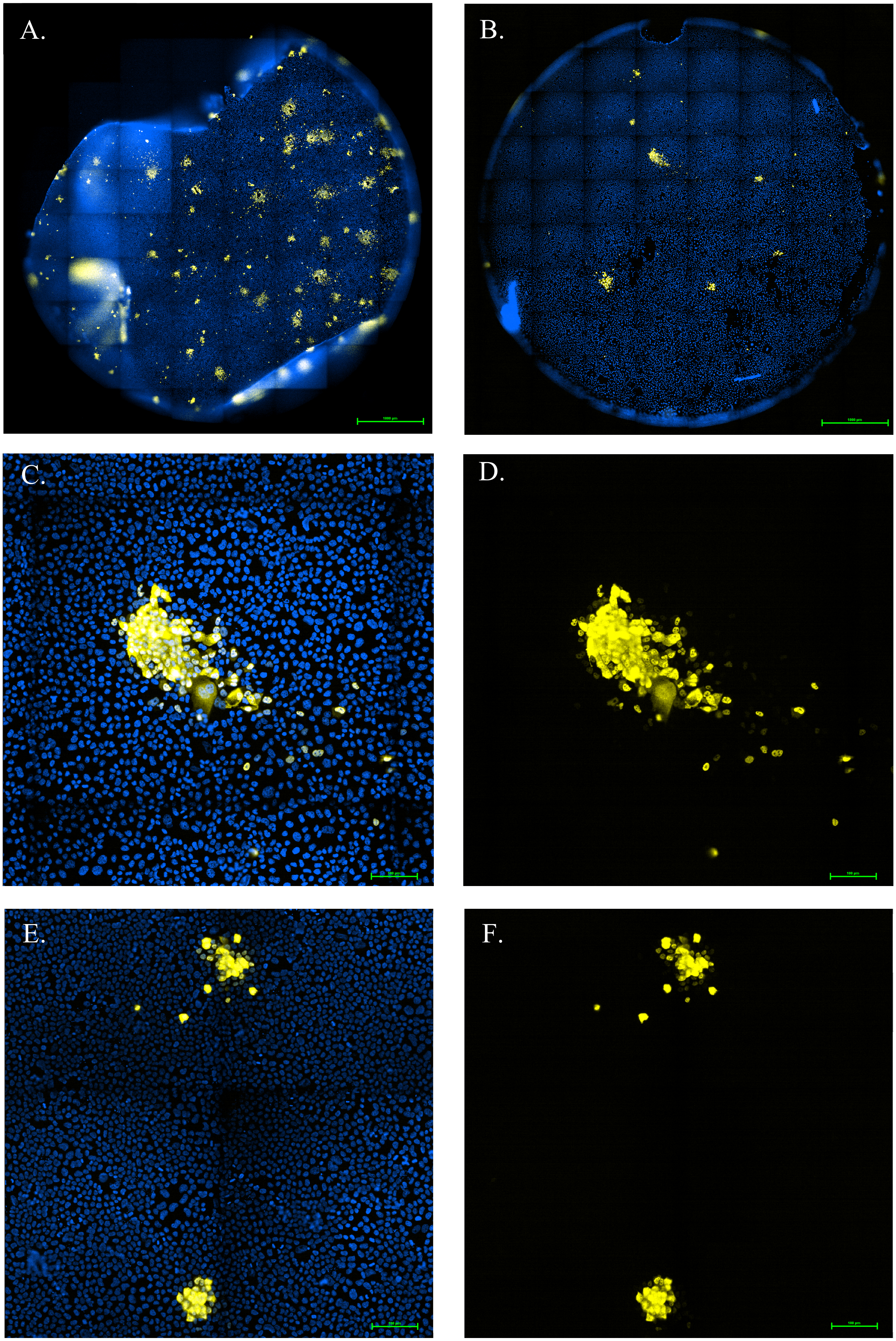
A.
B.
C.
D.
E.
F.

**Supplementary Figure S3.2.** Early proof of concept cluster-forming assay. (**A**) Monolayer peeling following exposure to cold (4 °C) reagents; always pre-warm reagents to between room temp and 37 °C before use. (**B**) Monolayer damage in low cell density areas; seed monolayers uniformly and at sufficient cell density. (**C, D**) PCU spillover. (**E, F**) PCU streak/comet. Yellow/YFP – A/Texas/50/2012 nucleoprotein ; Blue/Hoechst – MDCK-London nucleus.

**PCU spillover**: In a cluster-forming assay, *spillover* has occurred if there are satellite single-cell infection event(s) surrounding a PCU (**Supplementary Figure 3.2C-F, 3.3B-C**). Spillover undermines assay accuracy because it is unclear if these solitary infection events are progeny virus spawned by the closeby PCU, or a bona fide NCU from the initial inoculum. To mitigate PCU spillover, we pursued optimizations under certain key considerations. First, longer assay run times allow for farther diffusion of progeny virus from ground zero of a PCU infection. Second, less viscosity in the semi-solid overlay media increases flux of convection currents in the media, which also facilitates diffusion of progeny virus from ground zero of the PCU infection. As spillover was evident under conditions of 2% CMC and 19 hr assay runtime (**Supplementary Figure 3.2C-F, 3.3B-C**), we tested 8 hr, 10 hr, and 12 hr assay runtimes under 2% and 4% CMC. We found spillover to be mitigated under all tested conditions, however 8 hr and 10 hr were insufficient durations for PCUs to fully bloom and be counted as such, especially in slower growing strains like A/Texas/50/2012(H3N2) (**Supplementary Figure 3.4**). We also observed minor but inconsistent monolayer damage in the 2% CMC treatment groups, compared with no such damage in the 4% CMC groups. This damage was not observed during previous 19 hr assays, most likely because monolayer damage at the 12 hr time point had an additional 7 hr of recovery and resealing. Based on these findings, we settled on 4% CMC and 12 hr runtime for future assays involving A/California/07/2009(H1N1) and A/Texas/50/2012(H3N2) (**Supplementary Figure 3.5**).

**BIOHACKER ALERT**: Do not make CMC stock in microwave. Autoclave at 121°C for 30 min.

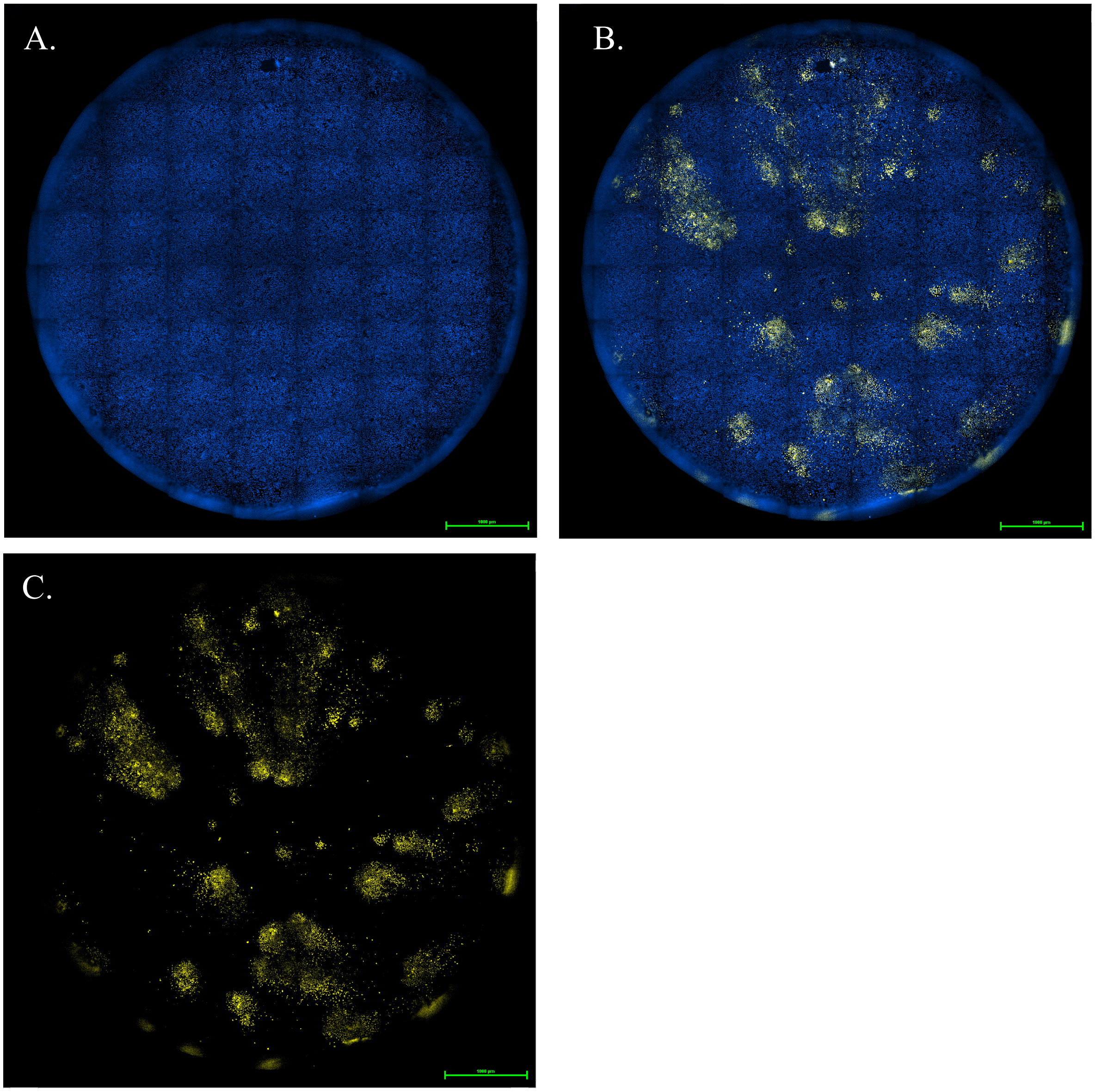


**Supplementary Figure S3.3.** (**A**) Barring aspirator pipette tip damage, a minimum cell seeding density (1E4 – 1.04E5 cells per well) is required to preserve monolayer integrity. However, PCU spillover and streaks still persist (**B-C**). Yellow/YFP – A/California/07/2009 nucleoprotein ; Blue/Hoechst – MDCK-London nucleus.

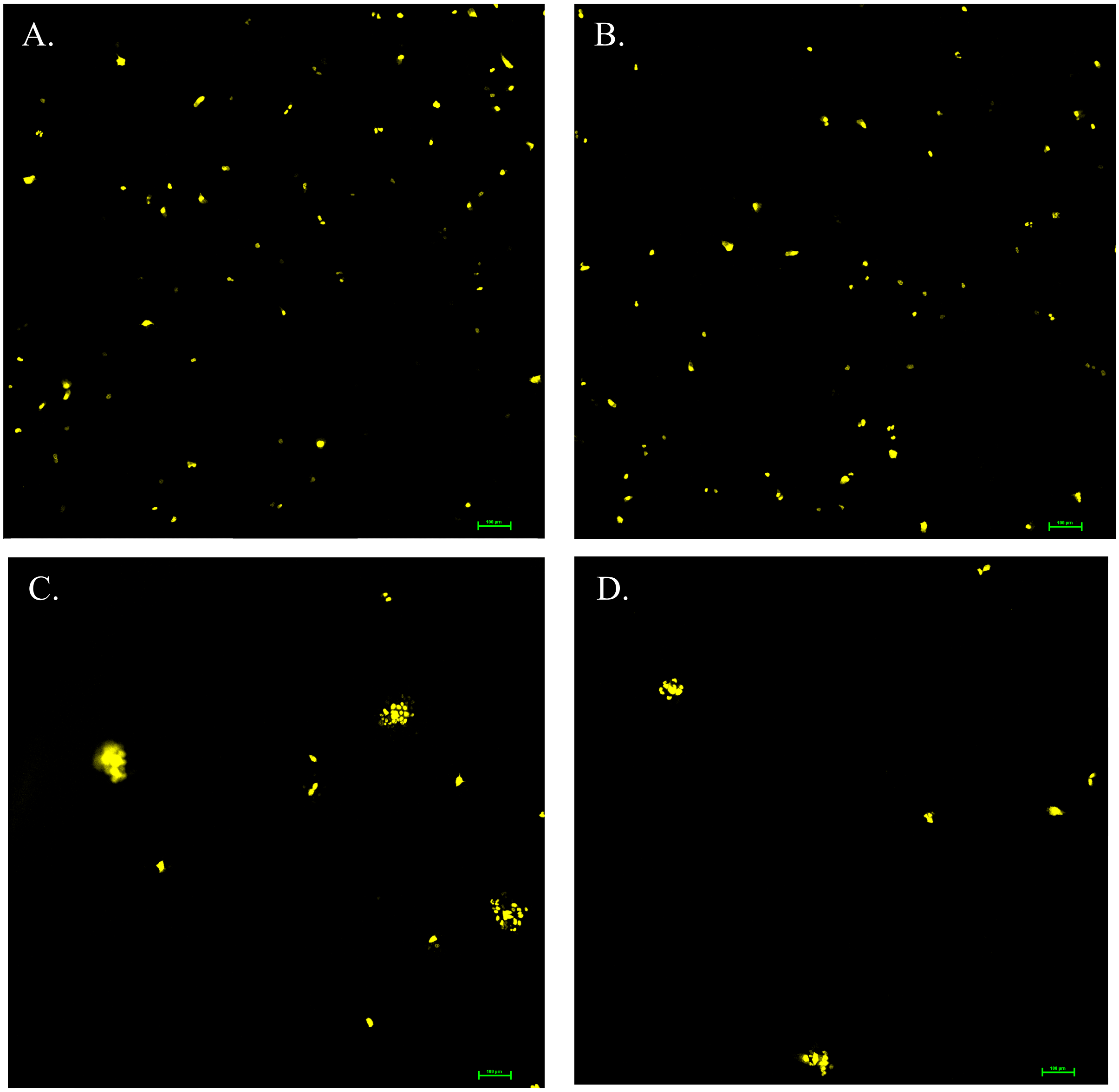


**Supplementary Figure S3.4.** Cluster-forming assay spillover mitigation; optimization of overlay concentration and assay runtime. (**A**) 8hpi at 2% CMC. (**B**) 8hpi at 4% CMC. (**C**) 12hpi at 2% CMC. (**D**) 12hpi at 4% CMC. Yellow(YFP) – A/California/07/2009 nucleoprotein.

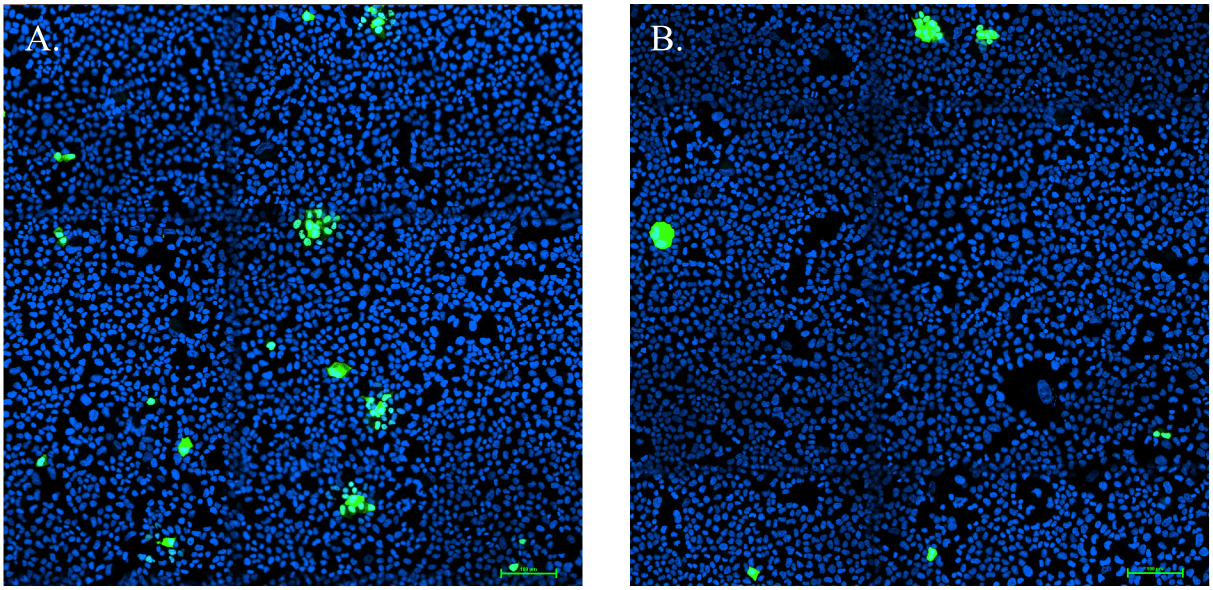


**Supplementary Figure S3.5**. Optimized cluster-forming assay; with damage-free monolayer and free of PCU spillover or streaks. Green/GFP – A/California/07/2009 nucleoprotein (**A**), A/Texas/50/2012 nucleoprotein (**B**) ; Blue/Hoechst – MDCK-London nucleus.

## *Cluster-counting Bioinformatics*

To count PCUs and NCUs, cluster-forming assay IF images (**Supplementary Figure 3.5**) were put through an automated image analysis pipeline we developed using MATLAB's image processing toolbox. Our guiding design principle was to cordon—or mask—nucleoprotein fluorescence signals (GFP) in the IF image as independent infection events, then overlay said mask with the host nuclei segmentation Hoechst signal to reveal the number of cells each infection event had spread to. We began stepwise assembly of masks around the GFP signals (**Supplementary Figure 3.6**) by binarizing IF images with the *imbinarize* function to make object detection possible, followed by the removal of small, noisy pixels with *bwareaopen*. Masks were sequentially dilated then filled with *imdilate* and *imfill* functions respectively to smoothen them out and ensure they did not contain holes. To finish the mask assembly, masks were eroded with *imerode* to undo the signal expansion done in the dilation step.

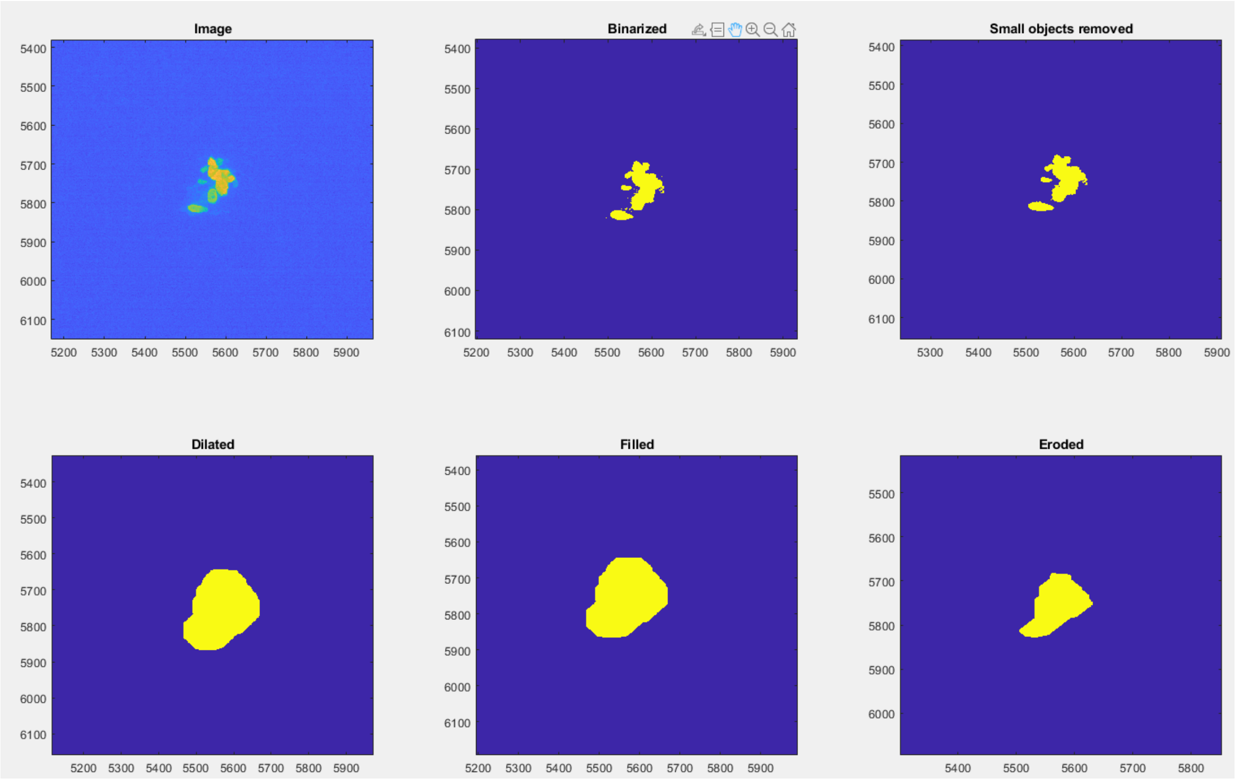


**Supplementary Figure S3.6.** Stepwise assembly of a mask around the nucleoprotein GFP signal in a productive clustering unit; starting from the initial cluster-forming assay IF image (**top-left**) down to final erosion (**bottom-right**).

At this juncture, the total number of masked objects in the binarized image included desired infection events, as well as undesired background noise from specs and autofluorescence. Undesired masks were filtered out by thresholding the min/max mask area and removing masks that did not contain any nuclei, leaving bona fide infection events—or clusters—that are counted and assigned a unique identity number (clusterID) (**Supplementary Figure 3.7**).

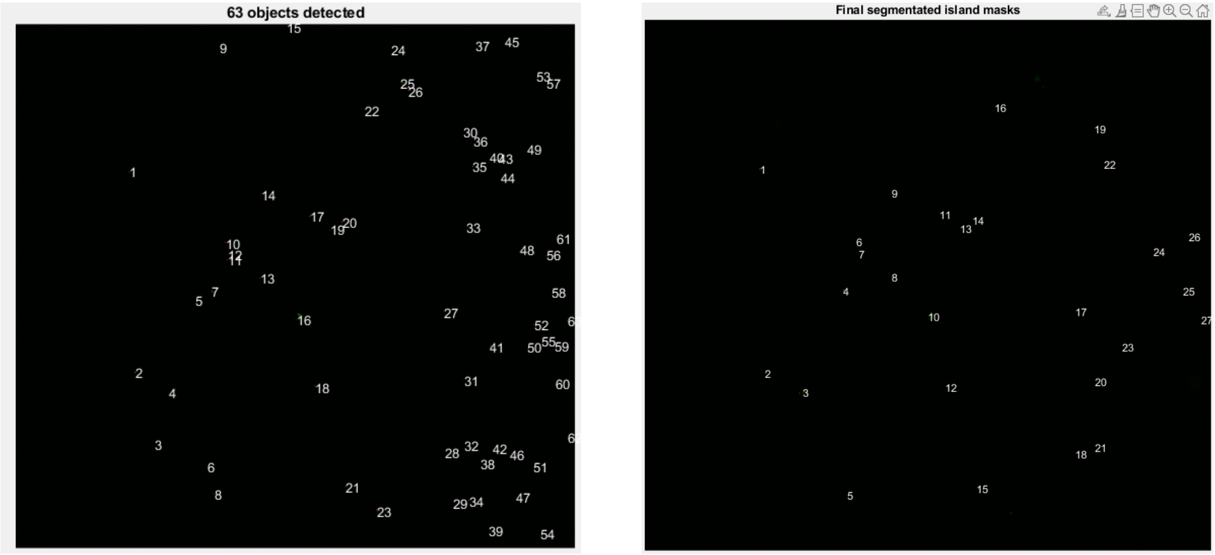


**Supplementary Figure S3.7.** Masked objects, before (**left**) and after (**right**) removal of background noise from specs and autofluorescence.

Next, segmentation was run on the Hoechst signal in host nuclei and overlaid with the masked clusters to demarcate and count the number of cells each infection had spread to (**Supplementary Figure 3.8**). Cluster data was sequentially exported to a spreadsheet then imported into the R Programming Language, where clusters were classified as PCU (ncell > 1) or NCU (ncell = 1), and after which NCU titer and NCU proportion were successively determined.

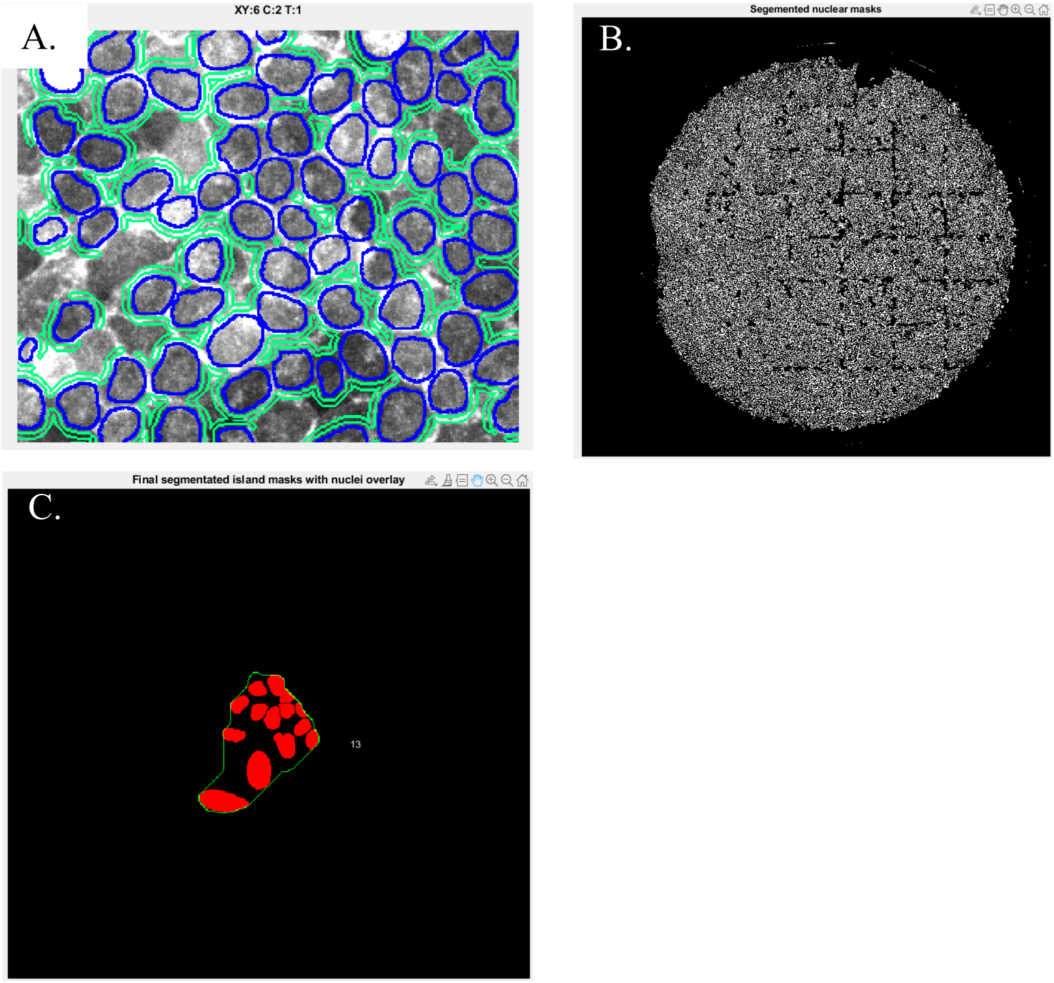


**Supplementary Figure S3.8.** **A.** Single-cell zoning of nuclear and cytoplasmic regions. Blue ring – nucleus; Green annulus – cytoplasm. **B.** Nuclear segmentation and **C.** the segmentation-mask overlay to size a clustering unit (PCU).
